# Development of a rapid in vitro pre-screen for distinguishing effective liposome-adjuvant delivery systems

**DOI:** 10.1038/s41598-022-14449-7

**Published:** 2022-07-20

**Authors:** Laura A. J. Feather, Vinod Nadella, Elisabeth Kastner, Yvonne Perrie, Anthony C. Hilton, Andrew Devitt

**Affiliations:** 1 College of Health and Life Sciences, Aston University, Birmingham, UK; 2 Strathclyde Institute of Pharmacy and Biomedical Sciences, University of Strathclyde, 161 Cathedral St, Glasgow, G4 0RE Scotland

**Keywords:** Innate immunity, Adjuvants

## Abstract

Liposomes are a strong supporting tool in vaccine technology, as they are a versatile system that not only act as antigen delivery systems but also adjuvants that can be highly effective at stimulating both innate and adaptive immune responses. Their ability to induce cell-mediated immunity makes their use in vaccines a useful tool in the development of novel, more effective vaccines against intracellular infections (e.g. HIV, malaria and tuberculosis). Currently, screening of novel liposome formulations uses murine in vivo models which generate data that often correlates poorly with human data. In addition, these models are both high cost and low throughput, making them prohibitive for large scale screening of formulation libraries. This study uses the cationic liposome formulation DDA:TDB (known as cationic adjuvant formulation 01 (CAF01)), as a lead formulation, along with other liposome formulations of known in vivo efficacy to develop an in vitro screening tool for liposome formulation development. THP-1-derived macrophages were the model antigen presenting cell used to assess the ability of the liposome formulations to attract, associate with and activate antigen presenting cells in vitro*,* crucial steps necessary for an effective immune response to antigen. By using a combination of in vitro functions, the study highlights the potential use of an in vitro screening tool, to predict the in vivo efficacy of novel liposome formulations. CAF01 was predicted as the most effective liposome formulation when assessing all in vitro functions and a measure of in vitro activation was able to predict 80% of the liposome correctly for their ability to induce an in vivo IFN-ү response.

## Introduction

Generally, in the development of all non-live vaccines, adjuvants are needed to enhance their efficacy. These adjuvants, when co-administered with antigen, elicit a good safety profile whilst helping to enhance the immunogenicity of the vaccine^1^. Adjuvants were the first compounds observed to induce a stronger immune response to sub-unit vaccines. This induction of a stronger immune response to the immunised antigen means the use of adjuvants can reduce the amount of antigen required in the vaccine, as well as the requirement for booster doses, thus reducing the cost of development and administration^2^. The use of adjuvants has also been found to increase the efficacy of vaccines in the immunocompromised, such as the elderly, newborns and HIV patients^3^. However, most current adjuvants used such as; alum, an oil-in-water emulsion MF59 and virosomes (used in influenza and Hepatitis A vaccines), all elicit limited cell-mediated responses^4,5^. Therefore there is an urgent need for adjuvants that induce a cell-mediated response. Developments have therefore been made to create more effective vaccines against pathogens that require a cell mediated response.

Within the past year the vaccine field has come leaps and bounds and has highlighted the ability to produce vaccines within a remarkably short time period against severe acute respiratory syndrome coronavirus 2 (SARS-CoV2). As part of this vaccine development programme against SARS-CoV2, 67 subunit and 15 inactivated vaccines are within development, all of which would require adjuvants to be able to induce a tailored immune response against COVID19^6^. Currently AS03, MF59 and CpG1018 are all licensed adjuvants (developed by GlaxoSmithKline, Seqirus and Dynavax), that have been made available for vaccine development against COVID19^7^. Valneva’s VAL2001 vaccine, against SARS-CoV2, is a vero-cell based, highly-purified inactivated whole virus vaccine which includes both Alum and CpG1018. CpG1018 is a toll-like receptor 9 agonist that has been licensed for use in the hepatitis B vaccine, HEPLISAV-B, where it was shown to increase antibody concentration, both CD4+ helper and CD8+ cytotoxic T cells, as well as robust T and B memory cells^8^. Whereas CpG1018 has shown to favour a Th1 immune response, Alum is known to favour a Th2/antibody response, therefore the combination of the two adjuvants target both arms of the adaptive immune system.

Two licensed vaccines against SARS-CoV2 (developed by Moderna and Pfizer) are mRNA vaccines incorporated within lipid nanoparticles^7^. Naked mRNA cannot be delivered without a delivery vehicle, as mRNA is sensitive to degradation by RNases and therefore lipid nanoparticles are used to promote protection and delivery of mRNA. In general, nanoparticles include, but are not limited to, lipid nanoparticles, virus-like particles and liposomes and can be used to enhance delivery to antigen presenting cells and also act as adjuvants in stimulating the innate immune system^9^.

Liposomes are synthetic, spherical vesicles composed of phospholipids, which create uni- or multi-lameller phospholipid bilayers, which were first described by Alec Bangham in the early 1960’s^10,11^. Liposomes have shown to be successful at both delivering antigen to target cells and enhancing the immunogenicity of vaccines, making them both delivery systems and immune stimulators^12^. The structural attributes of liposomes offers versatility in the type of antigen they can deliver; the hydrophilic core is efficient in delivering hydrophilic antigen, whilst the phospholipid bilayer creates a hydrophobic environment. The structure and type of the phospholipids that make up the liposome formulation also play a crucial part in the efficacy of liposomes. The polar-regions, attributed by the phosphate head group on the phospholipids, are responsible for the cationic, anionic or neutral charge of the liposomes. Whilst the non-polar hydrocarbon tails influence the properties of the lipid and therefore the liposome, including its transition temperature and bilayer fluidity according to the degree of saturation and length of the carbon chain. The different liposome properties have been shown to influence the type and strength of the immune response in vivo, in particular antigen retention, trafficking, uptake and processing by antigen presenting cells (APCs). Furthermore their ability to protect antigen from degradation helps to increase exposure time to APCs, often leading to an increase in antigen presentation to the adaptive immune system and a stronger resulting immune response^13,14^. Most importantly certain liposome formulations have shown to be good inducers of a cell-mediated, Th1 immune response, vital for infections such as TB and HIV^15^.

One of the most highly studied liposome adjuvant formulations is the cationic adjuvant formulation 01 (CAF01) consisting of the lipid dimethyldioctadecylammonium (DDA) and the immune-stimulatory component d-(+)-trehalose 6,6'-dibehenate (TDB). This liposome has been extensively studied pre-clinically and is now one of the few that have reached phase I clinical trials with the tuberculosis vaccine antigen Ag85B-ESAT-6 (H1)^16,17^. The constituent amphiphilic lipid DDA comprises of a positively charged dimethylammonium head group and two hydrophobic 18-carbon alkyl chain tails. Alone, DDA is relatively unstable with a tendency to aggregate over time. The addition of TDB, a synthetic analogue of the mycobacterial cell wall component trehalose 6,6′-dimycolate (TDM) acts to increase the adjuvanticity of the formulation through activation of the Mincle receptor on the surface of antigen-presenting cells (APCs), but also stabilises the liposomes and reduces spontaneous aggregation^18,19^.

As well as the addition of pathogen-associated molecular patterns (PAMPs) such as TDB, other adjuvant mechanisms of the CAF01 liposome, shown in vivo, include the formation of an antigen depot at the site of injection, improved antigen delivery to APCs, as well as causing local tissue damage to release damage associated molecular patterns (DAMPs) and increase cellular infiltration^20,21^. Pre-clinical studies investigating the immune response induced by CAF01, found the adjuvant to induce a combination of Th1 and Th17 responses, as well as a robust CD4 memory T cell response that was maintained beyond 1 year post-vaccination^22,23^. Furthermore, the CAF01-adjuvanted vaccine promoted the induction of more multifunctional CD4 T cells that accumulated at the site of Mycobacterium TB infection, compared to the current Bacillus Calmette–Guérin (BCG) vaccine^24^.

Despite these developments in liposome-adjuvanted vaccines, the speed of novel liposome formulation development and screening is inhibited by the in vivo screening process used in the pre-clinical phase. Currently screening of novel liposome formulations, to investigate their efficacy and to distinguish physiochemical characteristics that induce certain immune responses, is conducted using in vivo murine models. It involves investigating antigen deposition, bio-distribution and splenocyte differentiation, as well as cytokine and antibody profiles to establish the type of immune response induced. This results in a large number of animals being used; for just one liposome formulation approximately 10–50 mice could be required in order to assess antibody and cytokine responses, bio-distribution of the antigen, plus the number needed for controls. These numbers do not support the requirement for large-scale screening of liposomes; as for just 50 formulations, 250–500 mice would be required. As well as the use of mice in early stage screening, promising formulations would be taken further into guinea pigs and then larger animals before the liposome vaccine delivery system can move to human clinical studies. Furthermore, despite the fact that murine models have been used for hundreds of years within biomedical research, due to the similarities between the mouse and human immune system on a genetic level, there is still reduced reliability and reproducibility with animal models, biological differences and species variability which results in a limited number of liposome formulations progressing through to clinical studies^25^. There is also a high failure rate of liposome formulations in macaques monkeys due to data produced from mice not being comparable to responses in primates^26^. This process is therefore prohibitive in the use of animals and for high throughput screening of liposomes for more rapid development of vaccines and consequent benefits to human health.

To speed up the development of novel vaccines, a new way of liposome formulation screening, that can enable higher throughput and automation is required. By screening liposome formulations in the absence of antigen, the in vitro predictive model could aim to be used for all types of vaccines and infectious diseases, both human and non-human, allowing further adaption of the model dependent on what vaccine the liposome is to be used in. Furthermore, to increase reliability and reproducibility the use of animals in the process needs to be reduced, with the potential for full replacement. Therefore, this study looks at the use of a set of in vitro assays, which considers the required functions of an immune response, to be used as a rapid pre-screen for predicting potentially effective liposome formulations. The current study used liposomes of established in vivo function, in particular the use of the CAF01 formulation as an example of a lead formulation in order to determine the predictive power of the assays. The cationic liposome composition composed of DDA:TDB has been extensively investigated and a range of studies have previously investigated the impact of formulation^27,28^, stability^18^ and in vivo efficacy^29–31^. Therefore, we adopted these widely characterised liposomes in our studies. The human monocyte cell line, THP-1 cells were chosen for use as they allowed differentiation into macrophages when exposed to vitamin D3, the antigen presenting cell chosen to assess in vitro immune function of liposomes. Association of liposomes with THP-1-derived macrophages and chemotactic ability of the liposomes are assessed using flow cytometry and vertical cell migration assays. Whilst the ability of liposomes to activate APCs is conducted by cell phenotyping of co-stimulatory markers and release of inflammatory cytokines by enzyme-linked immunosorbent assays. The results shown here highlight the ability of a set of in vitro functions, consisting of the in vitro attraction of macrophages to liposomes, the in vitro association and the ability of liposomes to induce co-stimulatory marker expression and inflammatory cytokine release, to reveal functional differences between liposome formulations and to accurately predict which liposome formulations are the most effective in vivo.

## Materials and methods

### Cell culture and differentiation

Human THP-1 monocytes (obtained from ATCC; LGC Standards, Middlesex, UK) were cultured in ‘complete’ RPMI 1640 medium (Sigma Aldrich, UK) (10% (v/v) Foetal Bovine Serum (Gibco, UK), 1% Penicillin–Streptomycin and 1% L-glutamine solution (Sigma Aldrich, UK)) and incubated at 37 °C in 5% CO_2_ atmosphere with fresh medium added when a cell density of 5 × 10^5^–1 × 10^6^/ml was reached. To differentiate THP-1 monocytes into macrophage-like cells, THP-1 cells following centrifugation (300×*g*, 5 min) were resuspended in fresh complete RPMI 1640 medium at a density of 5 × 10^5^cells/ml. Cells were then stimulated with 100 nM dihydroxyvitamin D3 (VD3; Enzo Life Sciences, UK) and incubated at 37 °C for 48 h to allow differentiation into macrophage-like cells. Differentiation was confirmed by upregulation of cell surface CD14 (see Supplementary Fig. S1)^32^.

### Liposome production

Lipids (DDA (Dimethyldioctadecylammonium bromide), DSPC (1,2-distearoyl-sn-glycero-3-phosphocholine), DOTAP (1,2-dioleoyl-3-trimethylammonium-propane), DC-Chol (3ß-[N-(N',N'-dimethylaminoethane)-carbamoyl]cholesterol hydrochloride), and DSPE-PEG (1,2-distearoyl-sn-glycero-3-phosphoethanolamine-N-[amino(polyethylene glycol)-2000]) were purchased at 10 mg/ml in chloroform from Avanti Polar Lipids (Stratech Scientific Ltd, Ely, UK). TDB (D-(+)-trehalose 6,6'-dibehenate) was also purchased from Stratech Scientific and reconstituted in chloroform:methanol (9:1 v/v) at a concentration of 2 mg/ml. Cholesterol (chol) powder was purchased from Sigma Aldrich (UK) and dissolved in chloroform to 10 mg/ml. All lipids were stored at − 20 °C (Table 1).

**Table 1:** Composition of the liposome formulations tested within the in vitro assays.

Liposome formulation	Amount of lipid in 1 ml Tris buffer (mg)						
	DDA	DSPC	DOTAP	DC-Chol	DSPE-PEG2000	Cholesterol	TDB
DDA	1.25						
DDA:TDB	1.25						0.25
DDA:DSPC:TDB	0.625	0.625					0.25
DDA:DSPC:TDB (50%)	0.62	0.88					0.13
DSPC:TDB		1.25					0.25
DOTAP:TDB			1.38				0.25
DC-Chol:TDB				1.06			0.25
DDA:TDB:PEG25%	0.94				1.57		0.19
DDA:CHOL18%:TDB	1.25					0.19	0.25
DDA:CHOL31%:TDB	1.25					0.38	0.25

Table showing the mass of lipid (mg) in each of the liposome formulations to produce 1 ml of liposomes hydrated in 10 mM Tris buffer^28,33–35^.

Liposomes were produced and fluorescently labelled through the addition of 0.1 mol% 1,1'-Dioctadecyl-3,3,3',3'-Tetramethylindocarbocyanine Perchlorate (DiI C) (ThermoFisher Scientific, UK) using lipid film hydration (Kaur et al. ^34^). Briefly, lipids in the solvent phase were added together to a round bottomed flask at the appropriate ratios along with 0.1 mol % of DiI C. The solvent was then evaporated using a rotary evaporator (IKA RV 10) at 200 rpm for 6 min, leaving a dried lipid film on the bottom of the flask. Nitrogen was flushed over the lipid film to dispel any remaining solvent. The dried lipid film was rehydrated in 1 ml of 10 mM Tris (hydroxymethyl) aminomethane buffer (pH7.4; VWR- Analar NORMAPUR) buffer at 60 °C and vortexed. The lipid suspension was kept in a water bath above the transition temperature for 30 min and vortexed intermittently to produce multilameller liposomes.

### Characterization of liposomes

The size, polydispersity index and zeta potential of the liposomes were measured using dynamic light scattering (Brookhaven, Nanobrook 90Plus Zeta, SciMed Ltd). 100 µl of liposomes were diluted in 1.9 ml of 10 mM Tris Buffer (pH 7.4) and placed in a UV-grade square cuvette (Thermo Fisher Scientific). Size and polydispersity were measured using DLS size particle measurement. Zeta potential was measured using a palladium probe on the ELS Zeta Potential measurement with a conductance of ~ 1000 µS.

### Liposome association assay

Liposomes were produced via the lipid film hydration assay. DiI C (1,1'-Dioctadecyl-3,3,3',3'-Tetramethylindocarbocyanine Perchlorate) fluorescently-labelled liposomes were diluted to a concentration of 20 μg/ml in serum-free RPMI 1640 medium before being mixed at a 1:1 v/v ratio with VD3 differentiated THP-1 cells, that were resuspended in complete RPMI 1640 to a density of 2 × 10^6^ cells/ml. Co-culture was incubated at 37 °C in 5% CO2 atmosphere. At specific intervals (0, 5, 15, 30, 60, 90 and 120 min) 200 μl of co-culture was mixed with 200 μl of ice-cold serum-free RPMI before analysis. Interaction of fluorescent liposomes with differentiated THP-1 cells was analysed via flow cytometry (Beckman coulter FC500) 52 on the 488 nm laser (DiI C Ex-max, 549/Em-max 565 nm) using a minimum of 10,000 events per sample^36^. Analysis performed using FlowJo.

### Cell migration assay

Liposomes were diluted to a concentration of 20 µg/ml in serum-free RPMI 1640 medium, 700 µl of which was placed in the bottom of a 24 transwell ‘companion’ tissue culture plate (Falcon, Corning Life Sciences, UK). Differentiated THP-1 cells were re-suspended at a density of 2.66 × 10^5^ cells/ml in serum-free macrophage medium (Fisher, UK) and 300 μl were added to 8 μm transparent PET membrane cell culture inserts (Falcon, Corning Life Sciences, UK). The set up was sealed using the Cell IQ lid and adhesive tape before being connected to the Cell IQ system, with 5% CO_2_ gas enabled. Analysis of vertical macrophage migration was achieved by live cell imaging of the bottom surface of the transwell plate every 15 min for up to 18 h on the Cell IQ imaging system. Data was analysed using the Cell IQ analysis platform (CM Technologies, Finland).

### Flow cytometric phenotyping

Non-fluorescent liposomes were diluted to a concentration of 20 μg/ml in serum-free RPMI 1640 medium before being mixed at a 1:1 v/v ratio with VD3 differentiated THP-1 macrophages, that were resuspended in fresh complete RPMI 1640 to a density of 2 × 106 cells/ml. Co-culture was incubated at 37 °C in 5% CO_2_ atmosphere for 24 h. After incubation, 200 μl of each co-culture was placed into a 96 well, round bottom plate (10 wells per liposome-macrophage co-culture). The samples were centrifuged at 300×*g* for 5 min at 4 °C. The supernatant was discarded and 200 μl of 0.1% BSA in PBS was added to resuspend the cell pellet. Cells were washed by a repeat centrifugation at 300×*g* for 5 min again and removal of the supernatant to remove all RPMI medium. The addition of 200 μl of 0.1% BSA in PBS to wash the cells and centrifugation was repeated 3 times before phycoerythrin (PE)-conjugated antibodies were added to samples at a final Ab concentration of 1.25 μg/test (diluted in 0.1% BSA/PBS). Antibodies used; CD40, CD80, CD86, MHC II, MOPC21 and IgG2b (Invitrogen, UK). Once antibodies were added, the samples were incubated on ice, in the dark for 25–40 min. After incubation, 200 μl of 0.1% BSA/PBS wash buffer was added and samples were washed by centrifugation to remove unbound antibody (3 times). Samples were then fixed in 200 μl of 1% formaldehyde in PBS. Samples were analysed via flow cytometry (Ex-Max 565 nm/Em-Max 578 nm) with the forward scatter discriminator set to 100, using a minimum of 10,000 events per sample. Analyses were performed using FlowJo to determine the expression levels of each surface marker.

### Cytokine analysis

Samples of cell supernatant were prepared by incubating 20 μg/ml liposomes at a 1:1 v/v ratio with 1 × 10^6^/ml of VD3 stimulated THP-1 macrophages, at 37 °C for 24 h. 2 ml of the co-culture was centrifuged at 300×*g* for 5 min, the supernatant was collected and stored at − 80 °C and the cell pellet was discarded. Cytokine content of the cell supernatant were then assayed by ELISA. Following the manufacturer’s instructions from the DuoSet ELISA development kit, for cytokines IL-10, IL-12/IL23p40, IL-6, IL-1β/IL-1F2, IL-8/CXCL8 and TNF-α (R&D systems, UK), the sandwich ELISA was performed. The optical density of each sample was then determined using the MultiSkan Go (SkanIt RE 4.1) fluorescence plate reader (ThermoScientific) at 450 nm. The OD of the blank (capture and detection antibody with reagent diluent) was then subtracted from sample ODs to obtain an accurate OD reading.

### Statistical analysis

All data checked for normal distribution using a Kolmogorov Smirnov test. Normally distributed data was then analysed using one-way and repeated measures ANOVA with post hoc tests conducted using Graphpad prism software. Most often Tukey’s multiple comparison post hoc test used to compare the mean results from each liposome formulation. Differences were considered to be statistically significant at P < 0.05.

Principal Component Analysis was performed using XLSTAT plug in Excel (Microsoft Office). Raw data for in vitro association (% x mean fluorescence intensity (MFI)), migration (average cell count at 16 h), induction of surface marker expression (% x MFI) for CD14, CD16, CD11b, CD40, CD80, CD86, MHC II and cytokine release (concentration of TNF-α, IL-8 and IL-1β) was used to determine the relationship between in vitro functions and the liposome formulations tested. Results were displayed in a biplot on two principal components. Loading factors of the in vitro markers and PC scores of the liposome formulations showed any correlation between each of the in vitro markers, each of the liposome formulations and the relationship between the in vitro markers and the liposome formulations. Lines of origin were shown for the in vitro markers to allow interpretation of the relationship. Variables opposite to each other were determined as negatively correlated, positive correlation was determined with variables adjacent to each other, whilst a 90° angle highlighted no correlation. Liposome formulations were shown in groups according to correlations with each other and with the in vitro markers.

## Results

### Liposome characteristics

The 10 liposome formulations tested were split into two studies; study 1 consisted of DDA:TDB, DDA:DSPC:TDB, DDA:DSPC:TDB (50%), DSPC:TDB, DOTAP:TDB and DC-CHOL:TDB and these liposome formulations were tested and used for assay optimisation. The second study consisted of DDA, DDA:TDB:PEG25%, DDA:CHOL18%:TDB and DDA:CHOL31%:TDB. All liposomes were first characterised on the basis of their size, polydispersity index and zeta potential using dynamic light scattering (Table 2).

**Table 2:** Characteristics of all 10 liposome formulations produced by lipid-film hydration.

Liposome formulation	Mean size (nm) ± SEM	Mean PI	Mean ZP (mV)
DDA:TDB	592 ± 22	0.3	50
DDA:DSPC:TDB	851 ± 21	0.4	53
DDA:DSPC:TDB (50%)	1202 ± 29	0.4	58
DSPC:TDB	1758 ± 79	0.3	-15
DOTAP:TDB	553 ± 24	0.3	58
DC-CHOL:TDB	230 ± 5	0.3	45
DDA alone	453 ± 5	0.3	56
DDA:TDB:PEG25%	286 ± 2.1	0.3	20
DDA:CHOL18:TDB	613 ± 9.1	0.3	52
DDA:CHOL31:TDB	706 ± 8.2	0.3	51

Liposomes (DDA:TDB, DDA:DSPC:TDB, DDA:DSPC:TDB (50%), DSPC:TDB, DOTAP:TDB, DC-CHOL:TDB, DDA, DDA:TDB:PEG(25%), DDA:CHOL18:TDB and DDA:CHOL31:TDB) were produced via lipid film hydration, the lipid suspension was vortexed to produce multi-lamellar vesicles. Liposome characteristics; size (nm), polydispersity index (PI) and zeta potential (ZP) were measured using a Brookhaven ZetaPlus instrument. Results shown for n ≥ 3 (mean ± SEM).

The mean diameter of the liposomes showed that DSPC:TDB produced the largest liposomes > 1500 nm, whilst DC-CHOL:TDB produced the smallest liposomes at 230 nm, along with the PEGylated formulation that produced liposomes of 286 nm. DDA:TDB and DOTAP:TDB produced liposomes of similar size, 500–600 nm, with the addition of DSPC and cholesterol into the DDA:TDB formulation increasing the size of the liposomes. DDA:DSPC:TDB produced liposomes of 851 nm and DDA:DSPC:TDB (50%) produced liposomes of 1202 nm. Liposomes with the increasing addition of cholesterol also increased in size; DDA:CHOL18:TDB showed a mean diameter of 613 nm and DDA:CHOL31:TDB showed a mean diameter of 706 nm. Furthermore, the removal of TDB from the DDA:TDB formulation showed a reduction in liposome size, with the DDA liposome preparation showing a mean diameter of 453 nm.

The polydispersity index of all liposome formulations was approximately 0.3. The liposomes were also measured for their zeta potential using the ZetaPlus instrument. All liposome preparations gave a positive zeta potential, apart from DSPC:TDB that gave a negative zeta potential of − 15 mV. Aside from DDA:TDB:PEG25% and DC-CHOL:TDB that gave a zeta potential of 20 mV and 45 mV, the rest of the formulations gave a zeta potential between 50 and 58 mV.

### In vivo efficacy of the liposome formulations

To be able to develop a set of in vitro assays that assessed liposome function, and that may have the predictive power to establish different liposome formulation’s in vivo efficacy, the liposome formulations used had been previously tested in vivo for their potential as a vaccine adjuvant. DDA:TDB, is a liposome formulation that has been tested successfully in vivo, in murine models and also progressed through to human clinical vaccine trails^16^. This liposome formulation was therefore used as a positive control within all assays as an example of a formulation effective in vivo, and therefore any other liposome formulation tested would have had to have been tested alongside DDA:TDB in the in vivo studies.

In order to correlate the in vitro functions established from the in vitro assays, to the known in vivo efficacy of the liposome formulations, IFN-y production was chosen as the in vivo correlate of efficacy. Throughout this work in vivo IFN-ү production was chosen, as this is an established correlate of a Th1 immune response, though future studies could seek to compare to other correlates of immunity for particular pathogens of interest. With the emphasis of vaccine development being towards diseases such as tuberculosis (TB), HIV and malaria, it is important to establish the ability of vaccines to induce a protective cell-mediated, Th1 immune response. IFN-ү is a pro-inflammatory cytokine released from Th1 cells in response to IL-12. Th1 cells are required for cell mediated immunity and phagocyte-dependent inflammation, with most of their function being elicited by IFN-ү^37^. IFN-ү has a number of roles within the immune system; including, increased antigen presentation on major histocompatibilty complexes (MHC) I and II, IgG class switching, macrophage activation and increased phagocytosis, all of which help enhance the immune response towards vaccination^38^.

In order to establish the in vivo efficacy of the chosen liposome formulations, data from a set of in vivo studies, previously undertaken and reported, were selected that compared the liposome formulations to DDA:TDB and assessed the effect of the formulations on IFN-ү induction were used (Table 2).

Table 3 categorises the in vivo papers into study 1 and study 2, dependent on which liposome formulations they tested. Study 1 consists of three papers that investigate the adjuvant activity of DDA:TDB against, DDA:DSPC:TDB, DDA:DSPC:TDB (50%) and DSPC:TDB, as well as DOTAP:TDB and DC-CHOL:TDB. The tables show the amount of IFN-ү induced by each of the liposome formulations, from each of the three papers, as well as the calculated percentage of DDA:TDB of each of the responses. The effect of adding DSPC into the DDA:TDB formulation was investigated by Hussein et al. (2014). It was shown that IFN-Y production decreased from 4000 pg/ml when mice were immunised with DDA:TDB, to 2500 pg/ml with DDA:DSPC:TDB and 1500 pg/ml with a decrease in TDB within the formulation, DDA:DSPC:TDB(50%). When DDA was completely removed from the formulation to give DSPC:TDB, IFN-ү production decreased further to < 1000 pg/ml, which was not significantly different to when antigen alone was injected. The inability for DSPC:TDB to induce the production of IFN-ү was also observed in a study by Henriksen-lacey et al.^28^. Taking 4000 pg/ml as an 100% response, the other formulations could then be categorised for their in vivo efficacy compared to DDA:TDB. DDA:DSPC:TDB gave a response 63% of DDA:TDB and was therefore categorised as ‘++’ 50–79%, whilst DDA:DSPC:TDB (50%) and DSPC:TDB gave a response 38% and 25% of DDA:TDB so were both placed in the ‘+’ < 50% category. The in vivo efficacy of DOTAP:TDB and DC-CHOL:TDB was investigated by Henriksen-Lacey et al.^21^, here it was shown that both formulations only produced 20 ng/ml of IFN-Y, compared to 140 ng/ml from DDA:TDB and were therefore both categorised with in vivo efficacy of ‘+’.

**Table 3:** In vivo IFN-ү production induced by the chosen liposome formulations.

Study 1												
Liposome	IFN-γ production^1^	IFN-γ production (% of DDA:TDB)	In vivo strength	IFN-γ production^2^	IFN-γ production (% of DDA:TDB)	In vivo strength	IFN-γ production^3^	IFN-γ production (% of DDA:TDB)	In vivo strength			
DDA:TDB	~ 4 ng/ml	100%	+++ > 80% (> 3.2 ng/ml)	140 ng/ml	100%	+++ > 80% (112 ng/ml)	> 20 ng/ml	100%	+++ > 80% (16 ng/ml)			
DDA:DSPC:TDB	~ 2.5 ng/ml	63%	++50–79% (> 3.2 ng/ml)									
DDA:DSPC:TDB (50%)	~ 1.5 ng/ml	38%	+ < 50% (< 2 ng/ml)									
DSPC:TDB	< 1 ng/ml (NS to Ag alone)	25%	+ < 50% (< 2 ng/ml)				< 5 ng/ml (NS to Ag alone)	25%	+ < 50% (10 ng/ml)			
DOTAP:TDB				20 ng/ml	14%	+ < 50% (70 ng/ml)						
DC-Chol:TDB				20 ng/ml	14%	+ < 50% (70 ng/ml)						

Study 2												
Liposome	IFN-γ production^1^	% of DDA:TDB	In vivo strength	IFN-γ production^2^	% of DDA:TDB	In vivo strength	IFN-γ production^3^	% of DDA:TDB	In vivo strength	IFN-γ production^4^	% of DDA:TDB	In vivo strength
DDA:TDB	~ 1.8 ng/ml	100%	+++ > 80% (1.44 ng/ml)	~ 1.6 ng/ml	100%	+++ > 80% (1.28 ng/ml)	~ 110 ng/ml	100%	+++ > 80% (88 ng/ml)	~ 100 ng/ml	100%	+++ > 80% (80 ng/ml)
DDA alone							~ 20 ng/ml	18%	+ < 50% (55 ng/ml)	~ 20 ng/ml	20%	+ < 50% (50 ng/ml)
DDA:TDB:PEG25%	~ 0.5 ng/ml	28%	+ < 50% (< 0.9 ng/ml)									
DDA:CHOL18:TDB				~ 1.2 ng/ml	75%	++50–79% (0.8 ng/ml)						
DDA:CHOL31:TDB				~ 0.8 ng/ml	50%	++50–79% (0.8 ng/ml)						

The tables below show the amount of IFN-ү produced from splenocytes of mice, after immunisation with a TB vaccine antigen, adjuvanted with the discussed liposome formulations. Each of the papers investigated the immune efficacy of the liposome formulations alongside DDA:TDB, therefore an IFN-ү production as a percentage of DDA:TDB was calculated for each of the liposomes within each paper. In order to calculate a percentage of DDA:TDB result, responses induced by DDA:TDB were taken as 100%, the responses of the other liposome formulations were then calculated as a percentage of the DDA:TDB response. The percentages were categorised into three levels of in vivo efficacy; strong efficacy ‘+++’ > 80% of DDA:TDB, intermediate efficacy ‘++’ 50–79% of DDA:TDB and weak efficacy ‘+’ < 50%. Study 1 references: ^1^Hussain et al.^33^. ^2^Henriksen-Lacey et al.^21^. ^3^Henriksen-lacey et al.^28^. Study 2 references: ^1^Kaur et al.^34^; ^2^Kaur et al.^35^; ^3^Henriksen et al.^21^; ^4^Davidsen et al.^18^.

In study 2, the addition of PEG into the DDA:TDB formulation was investigated by Kaur et al.^34^, were they showed a 72% reduction in IFN-ү produced. The addition of cholesterol was also shown to reduce the amount of IFN-ү produced in response to the immunised antigen. DDA:CHOL18:TDB produced 1200 pg/ml compared to 1600 pg/ml from DDA:TDB, whilst DDA:CHOL31:TDB produced 800 pg/ml^35^. Furthermore, when investigating the adjuvant activity of DDA alone, both Henriksen et al.^21^ and Davidsen et al*.* (2005) observed a 80–82% reduction in IFN-ү production with DDA compared to DDA:TDB.

**Figure 1:**
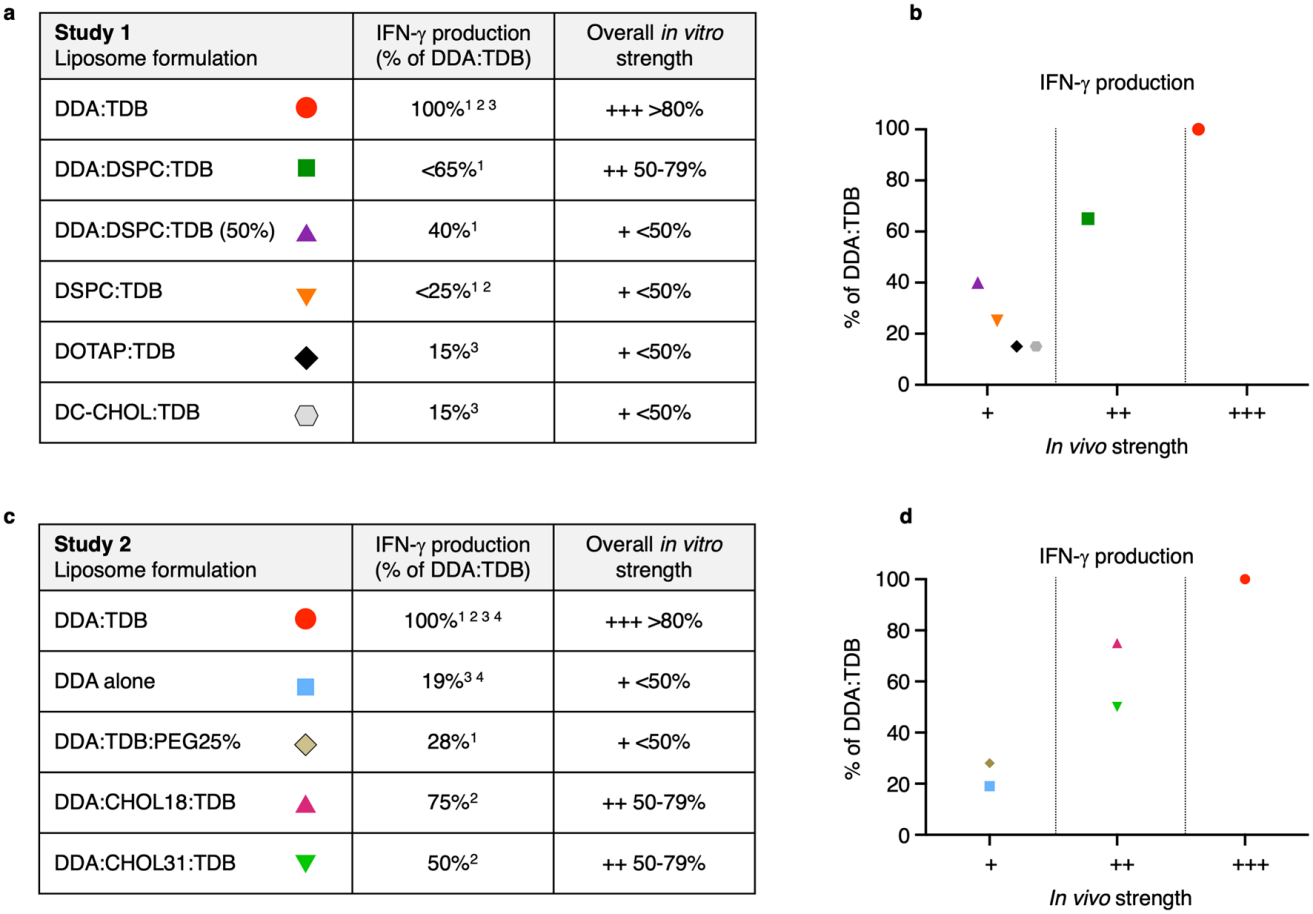
In vivo IFN-ү production induced by exposure to liposome formulations, compared against DDA:TDB provides in vivo liposome efficacy. Data from in vivo papers referenced within the figure were taken for each liposome formulation. (**a**) Shows a summary table combining data, from all study 1 papers, of IFN-ү production shown as the percentage of DDA:TDB, and the corresponding in vivo strength category established as ‘+++’ > 80%, ‘++’ 50–79% and ‘+’ < 50%. (**c**) Shows a summary table combining data, from all study 2 papers, of IFN-ү production shown as the percentage of DDA:TDB, and the corresponding in vivo strength category established as ‘+++’ > 80%, ‘++’ 50–79% and ‘+’ < 50%. This data is represented in (**b**) and (**d**). Study 1 references: ^1^Hussain et al.^33^. ^2^Henriksen-Lacey et al.^21^. ^3^Henriksen-lacey et al.^28^. Study 2 references: ^1^Kaur et al.^34^; ^2^Kaur et al.^35^; ^3^Henriksen et al.^21^; ^4^Davidsen et al.^18^.

Figure 1 summarises the in vivo efficacy of the liposome formulations used in both study 1 and study 2. The collection of in vivo studies for all the 10 formulations studied, highlighted DDA:TDB was the most effective formulation at inducing IFN-ү in vivo, whilst the other 9 formulations gave lower responses. All formulations were either categorised as ‘++’ 50–79% or ‘+’ < 50%.

### In vitro migration of macrophages towards liposomes

**Figure 2:**
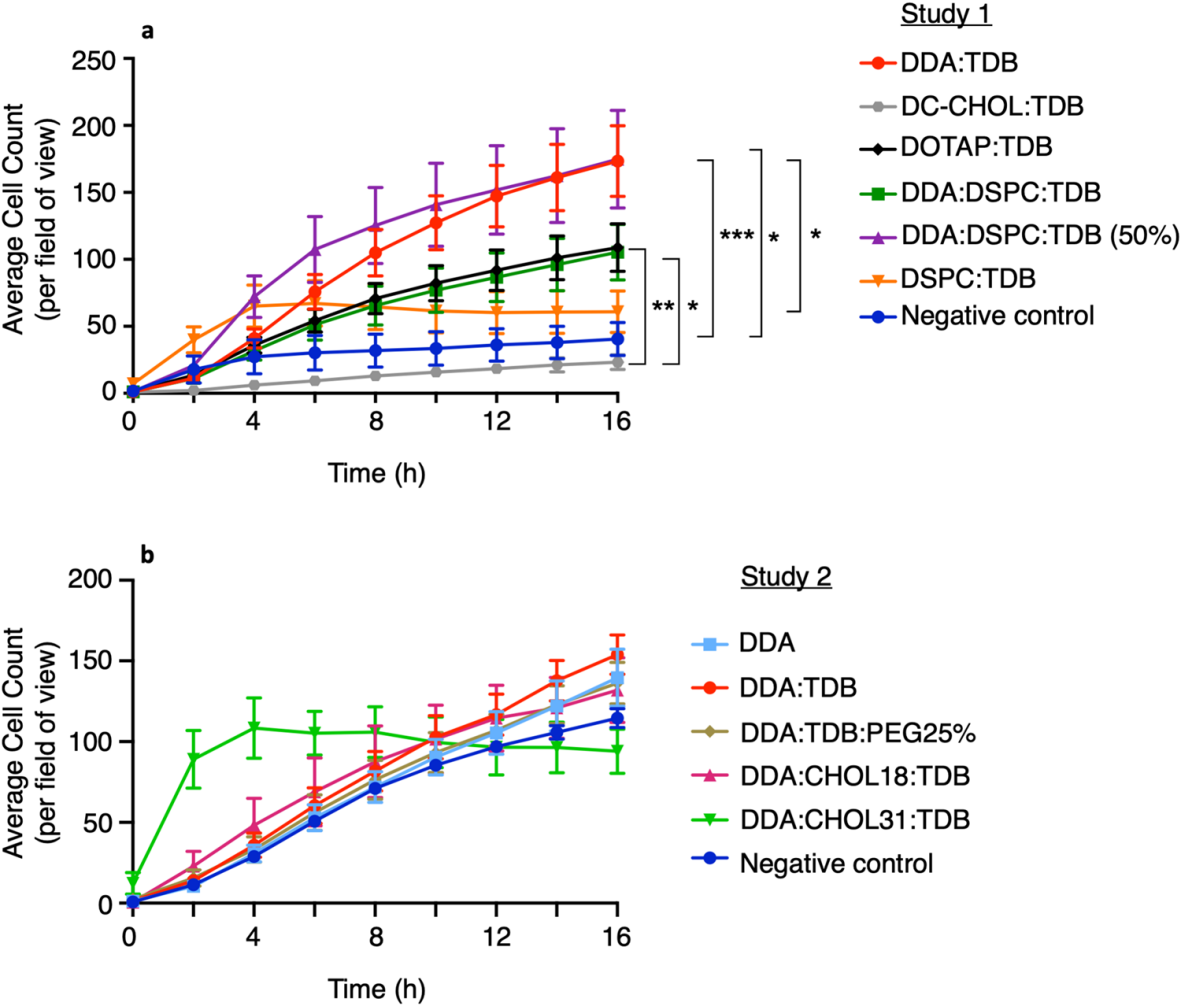
The extent of migration of THP-1-derived macrophages towards liposomes is dependent on the formulation. 700 µl of liposomes diluted to 20 µg/ml in serum-free RPMI were placed in the bottom well of a 24-well transwell culture plate and 300 μl of VD3-stimulated macrophages, at a cell density of 2.66 × 10^5^ cells/ml placed in an 8 µm pourable insert above. The plate set up was incubated at 37 °C for over 16 h within the Cell IQ live cell imager. Images were taken every 2 h to enable the number of macrophages that had migrated towards the liposomes to be counted. (n ≥ 3 mean ± SEM). *P ≤ 0.05, **P ≤ 0.005, ***P ≤ 0.0005, ****P ≤ 0.0001 with repeated measures two-way ANOVA and Tukey’s multiple comparison test.

In order for quiescent APCs to interact with and be activated by liposomes, they first need to be attracted to and/or recognise the liposomes. We hypothesised that the most in vivo active liposomes would be the most attractive to APCs. To assess this, an in vitro vertical cell migration assay was used, in which the panel of liposomes was placed in a lower well of a transwell culture plate and macrophages were placed in 8 µm transwell upper chamber. Images of the lower chamber were taken using the Cell IQ live cell imager over a 16 h time period and cells were counted using the Cell IQ analysis platform (Fig. 2).

Figure 2a shows that for all liposome formulations, except for DSPC:TDB, a steady increase in migrating cell count was noted over the 16 h period, reaching a maximum cell count at 16 h. Migration towards DSPC:TDB however, reached a maximum cell count of ~ 60 at 4 h, then plateaued over the next 12 h, attracting a number of cells not significant from the negative control (38 cells) of serum-free RPMI at 16 h. At 16 h (where maximum migration occurred) revealed that only DDA:TDB and DDA:DSPC:TDB (50%) attracted a total number of macrophages significantly (p < 0.05) higher than the negative control, of 173 and 174 respectively. DOTAP:TDB and DDA:DSPC:TDB showed the same pattern of attraction and both attracted ~ 100 macrophages at 16 h, which was neither significantly different from the negative control nor DDA:TDB. DC-CHOL:TDB was the liposome formulation to attract the fewest macrophages (~ 20) in study 1 and therefore did not induce significant migration capacity in the macrophages.

Figure 2b, shows results from liposome formulations in study 2. Results show the same steady increase in the number of macrophages to have migrated towards the liposomes, as observed in study 1. DDA:TDB attracted the most macrophages, with an average of 154 macrophages at 16 h, whilst DDA alone attracted 140, DDA:TDB:PEG25% attracted 136 and DDA:CHOL18:TDB attracted 132. However the number of macrophages that had migrated towards each of the liposome formulations was not significantly different to the negative control of serum-free RPMI, which saw 115 macrophages migrated over the 16 h. In contrast to the continuous increase in migration observed with the other liposome formulations, DDA:CHOL31:TDB induced a more rapid increase in migration, where the maximum number of macrophages to have migrated was reached at 4 h. Migration than plateaued and the number of macrophages to have migrated stayed the same over the next 12 h.

The migration data clearly reveal different migration inducing capacity of the different liposome formulations, and in both studies, DDA:TDB induced migration in the highest number of macrophages. The in vitro migration assay was also able to highlight those formulations that were not effective at inducing migration in macrophages, particularly DSPC:TDB and DC-CHOL:TDB.

### Association of liposomes with macrophages

Previous in vivo murine work using DDA:TDB liposomes indicates a Th1 immune response is induced in response to challenge^16^. The induction of an adaptive immune response, such as a Th1 response, suggests cellular uptake of the liposomes most likely by antigen presenting cells (APCs) and APC activation in order to elicit a T cell effector and memory response^24^. To assess if this response could be modelled in vitro*,* the necessary steps for the induction of an immune response and the development of immunological memory were exploited for study. Firstly, APCs need to be able to recognise and associate with antigen via its delivery system (liposomes in this case), the APCs then need to become activated in order to present antigen and activate the adaptive immune response. With antigen uptake being a crucial step in initiating an immune response, in vitro liposome association with THP-1-derived macrophages was assessed using flow cytometry. Macrophages were chosen as an easily induced and robust cell system^32^, that is known for PAMP-responsiveness and high phagocytic ability compared to dendritic cells, as well as their ability to activate non-naïve T cells and influence B cell activation and class switching^29,39^. Furthermore, these macrophage-like cells are lightly adherent and easily amenable to the assays used here.

**Figure 3:**
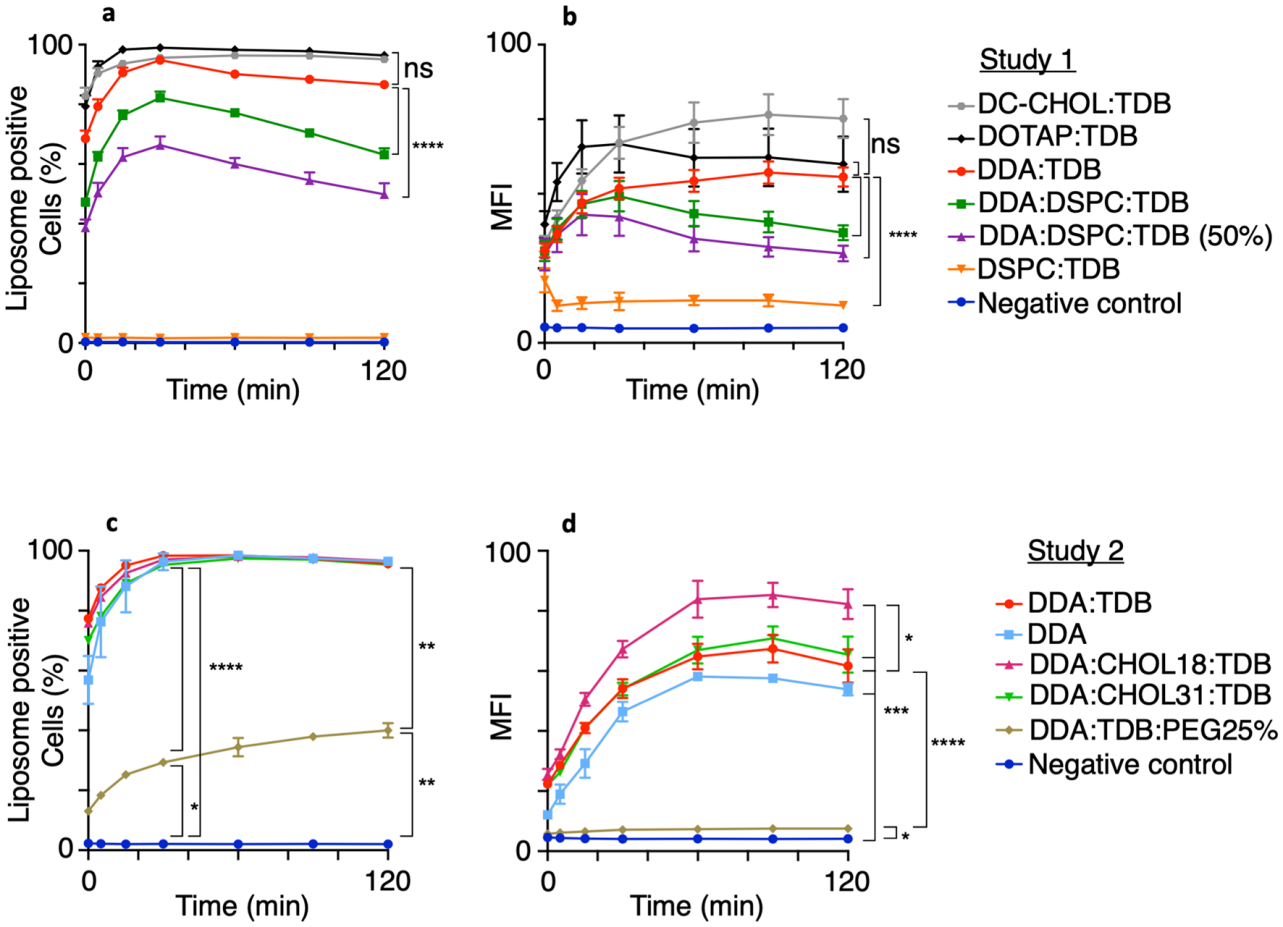
In vitro macrophage uptake of liposomes can reveal functional differences between formulations. Liposomes were diluted to 20 μg/ml in serum-free RPMI co-cultured 1:1 with VD3- stimulated macrophages at a density of 1 × 10^6^/ml and incubated for 2 h. At indicated time points, 200 µl of co-culture was mixed with 200 µl ice cold sfRPMI medium before flow cytometric analysis of 10,000 events. (**a**) and (**c**) show percentage of macrophages positive for liposome association and (**b**) and (**d**) show the mean fluorescence intensity from cells positive for liposome association. Data shown for n = 3 (mean+/- SEM) with repeated measures ANOVA and Bonferroni post hoc test at 30 min, *P ≤ 0.05, **P ≤ 0.005, ***P ≤ 0.0005, ****P ≤ 0.0001. (Flow histograms shown in Supplementary Figs. S2 and S3).

In order to assess liposome association with macrophages, the liposomes were fluorescently labelled with DiI C and co-cultured with THP-1-derived macrophages. At various times post-co-culture, samples were taken for flow cytometric analysis of liposome-macrophage association (Fig. 3). It was hypothesised that those liposome formulations that attracted the most macrophages, would be the formulations that associated with macrophages most effectively.

Figure 3a,c show the percentage of macrophages associated with fluorescent liposomes in a time-dependent manner. Assessing formulations that changed the content of the cationic lipid DDA and the neutral lipid DSPC in study 1 (Fig. 3a), DDA:TDB liposomes associated with the highest percentage of macrophages, reaching ~ 90% at 30 min of incubation. The addition of DSPC into the DDA:TDB formulation led to a significant decrease in liposome-macrophage association; with DDA:DSPC:TDB associating with ~ 80% at 30 min and DSPC:TDB associating with macrophages no better than the negative control of macrophages alone. When taking the DDA:DSPC:TDB formulation and reducing the amount of TDB by 50%, there was a further decrease in uptake to ~ 65% at 30 min. Interestingly, the formulations without DDA; DOTAP:TDB and DC-CHOL:TDB, interacted with ~ 90–100% of the cell population at 30 min, which highlighted strong in vitro association comparable to DDA:TDB.

Figure 3c shows that all liposomes in study 2, aside from DDA:TDB:PEG25%, were very efficient at associating with macrophages, as at 30 min when maximum association was achieved, nearly 100% of the cell population had associated with the liposome formulations. The percentage of macrophages to have associated with the liposome formulations was not significantly different between each of the formulations, as DDA:TDB associated with 98%, whilst DDA:CHOL18%:TDB associated with 97%. Furthermore, DDA alone associated with 96% of cells at 30 min and DDA:CHOL31%:TDB associated with 95%. DDA:TDB:PEG25%, however, only associated with 30% of the macrophage population, which was significantly lower than the association seen with the other formulations. However, whilst association with the other liposome formulations started to plateau at 30 min, the number of macrophages that had associated with DDA:TDB:PEG25% continued to slowly increase until it reached association with 40% of macrophages at 120 min. The most significant difference between the liposomes and the negative control of macrophages alone was seen at 30 min, when maximum association was reached.

Results in Fig. 3b,d show the mean fluorescence intensity (MFI) of macrophages that had associated with Dil-C-labelled liposomes (relative number of liposomes associated per cell). The trend for each of the liposomes was the same as that seen in Fig. 3a, where liposomes that had associated with the highest percentage of macrophages also elicited the highest MFI. The MFI results in Fig. 3d appeared to be able to differentiate between the various liposome formulations more efficiently than the percentage association results. The results suggest that more DDA:CHOL18:TDB liposomes associated with each macrophage, as macrophages within this population elicited a maximum MFI of 4 at 120 min. Furthermore, DDA:CHOL31:TDB and DDA:TDB elicited a similar MFI of 3, whereas DDA alone elicited a slightly lower MFI of 2.7 and DDA:TDB:PEG25% of 0.4, all significantly higher than the MFI obtained from the negative control of 0.2.

These results suggest that the liposomes associated with macrophages almost immediately and that when 100% of the macrophage population had associated with the liposomes, if there were still liposomes that had not associated, they would continue to associate with macrophages. This in turn increased the MFI results and helped to further differentiate between the in vitro association efficacies of the liposome formulations. This immediate interaction could be due to electrostatic interactions between the macrophages and liposomes. Studies conducted with DDA:TDB at 4 °C, 19 °C and 37 °C showed that whilst there was a significant decrease in association at 4 °C, suggesting some of the association was by phagocytosis, 74% association still occurred, suggesting electrostatic interactions were also present (Supplementary Fig. S4).

### Altering the size of DSPC:TDB does not influence in vitro cellular association with macrophages

Previous studies have shown that vesicle size of DDA:TDB influences in vivo responses with liposomes of ~ 500 nm promoting a higher IFN-ү response from splenocytes than large (> 1000 nm) and small (~ 200 nm) liposomes^29^. However, it was also noted that larger liposomes induced the highest level of splenocyte proliferation and thus were significantly better at inducing memory T cells. Other studies have supported these results and found that small unilamellar DDA:TDB liposomes of ~ 500 nm, containing surface-adsorbed OVA antigen, significantly increased both spleen CD8 and CD4 IFN-y responses compared to larger multilamellar liposomes (> 1000 nm)^30^.

**Figure 4:**
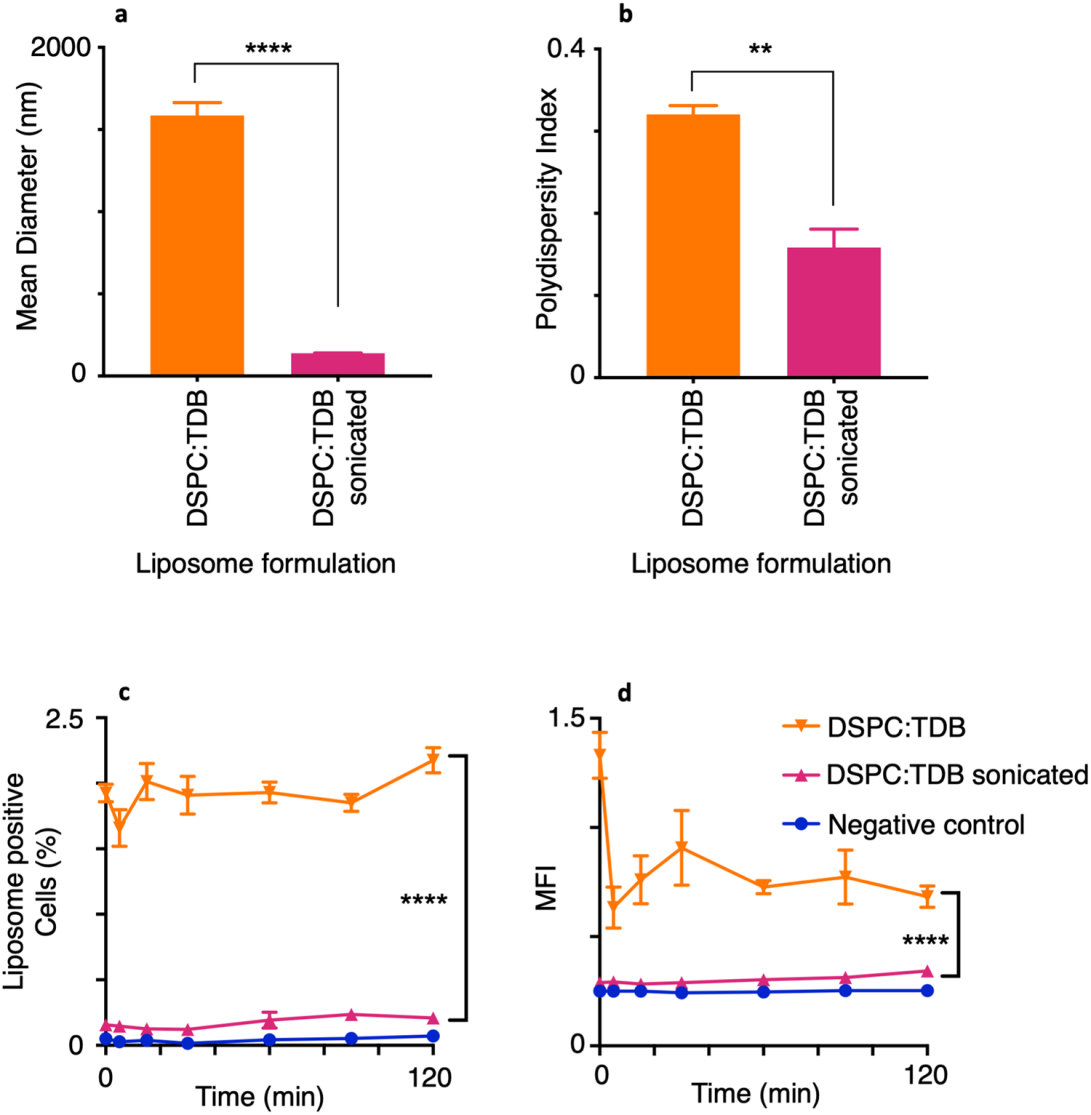
Altering the size of DSPC:TDB does not influence in vitro cellular uptake. DSPC:TDB liposomes produced via lipid film hydration to make large (> 1000 nm) liposomes. Large liposomes then sonicated at 45 °C for 15 cycles using a Diaganode water bath sonicator to produce small (~ 200 nm) DSPC:TDB liposomes. Liposome characteristics; size (**a**) and polydispersity (**b**) of both sonicated and non-sonicated liposomes were measured using dynamic light scattering (Brookhaven ZetaPlus). THP-1-derived macrophages at 2 × 10^6^/ml were co-cultured 1:1 with DilC-fluorescently labelled liposomes at 20 μg/ml. At indicated time points, 200 μl of co-culture was analysed using flow cytometry (10 000 events) for percentage of macrophages positive for association (**c**) and mean fluorescence intensity of macrophages (**d**). (n = 3, mean ± SEM) (**a**) and (**b**) show significance of ****P ≤ 0.0001 with a multiple T test and (**c**) and (**d**) show significance ****P ≤ 0.0001 with repeated measures ANOVA and Bonferroni post hoc at 30 min.

With size having an influence on various aspects of the in vivo response, it is possible that various results presented in Fig. 3 were due partly to liposome size, with smaller liposomes associating most with macrophages. Therefore, the effect of liposome size on in vitro association by THP-1 macrophages was investigated. DSPC:TDB was chosen for detailed study as this formulation produced the largest liposomes of > 1000 nm and showed no significant association with macrophages. In order to assess if reducing liposome size promoted liposome-macrophage association, DSPC:TDB liposomes were produced, as described previously, via lipid film hydration. This was then split into two batches, with one batch having a size of > 1000 nm and the remainder sonicated to produce liposomes of a reduced size of 138 ± 5 nm and a PDI of ~ 0.15, significantly different from un-sonicated liposomes (Fig. 4a,b).

To assess association with macrophages, the different sized-liposome preparations were then co-cultured with THP-1-derived macrophages, to assess liposome association as before. These studies revealed that the reduction in size of DSPC:TDB liposomes did not improve association with macrophages, either as measured by percentage association or MFI (Fig. 4c,d) and instead decreased the percentage of macrophages associated significantly from 2 to 0%. The same reduction was noted with MFI (Fig. 4d). These results suggest that the initial factor in influencing in vitro and potentially in vivo liposome function is lipid content rather than liposome size and consequently only when the lipid content is favourable for association, may size of liposome have a subsequent influence. This is a promising observation for the in vitro association assays studied here, as it suggests that results are not biased by changing liposome size, unless the lipid composition is favourable in the first place to enable APC-association.

### Expression of co-stimulatory markers on macrophages exposed to different liposome formulations

Having established the capacity for liposomes to induce migration in macrophages, as well as the ability of macrophages to associate with liposomes, the next step was to establish the ability of liposomes to induce an activation state in the macrophages. In order for an immune response to be initiated against the pathogen/vaccine, APCs have to communicate with the lymphocytes of the adaptive immune system. One of the methods of communication is the expression of certain surface markers and therefore activation was assessed by measuring expression of co-stimulatory markers on the surface of the macrophage.

CD80, CD86 and CD40 are co-stimulatory markers required for T cell activation upon antigen recognition. CD40 is a receptor known to be expressed on APCs, which binds to the CD40L ligand expressed on activated T cells, this binding is required for both macrophage maturation and T cell priming^40^. It is important that liposomes are able to induce expression of CD40, as without CD40-CD40L binding APC maturation, release of inflammatory cytokines and certain T cell responses would be defective^41^. Furthermore, CD86 and CD80 on the surface of APCs bind to CD28 on the surface of T cells, acting as a co-stimulatory signal to augment T cell function^42^. The action of co-stimulatory molecules acting as a second signal to complete T cell activation occurs alongside antigen recognition of the T cells cognate antigen, presented on MHC II on APCs. MHC II is a cell surface receptor that allows for presentation of the processed antigen to T cells, in which its expression levels will augment a Th1/Th2 skew^43^. With the importance of co-stimulatory molecules and the ability of APCs to present antigen to T cells via MHC II molecules, it was hypothesised that liposomes known to be effective in vivo at stimulating the adaptive immune system (and inducing IFN-ү production), would increase surface expression of the molecules in vitro.

**Figure 5:**
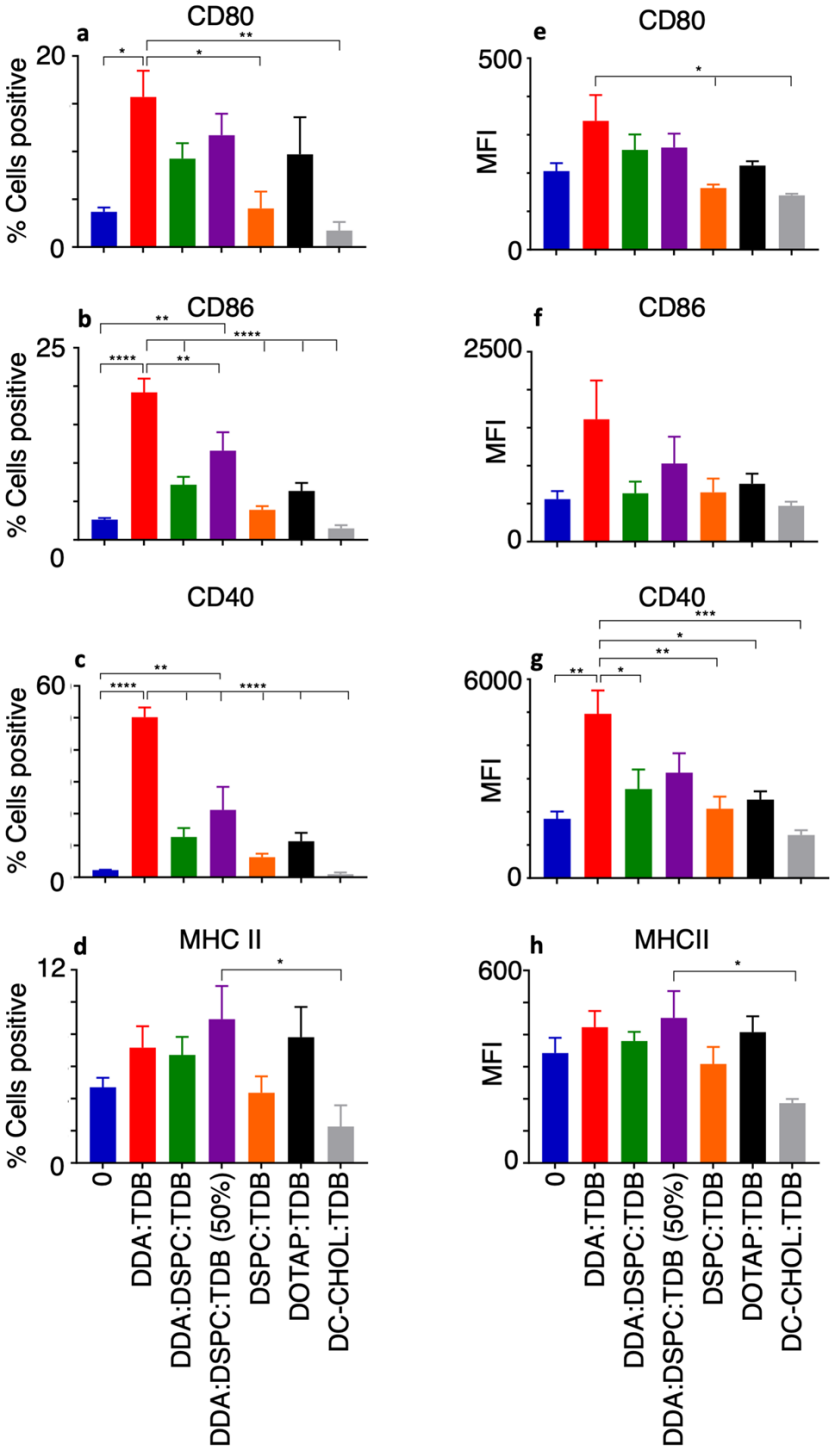
Study 1: Assessing changes in surface marker expression from VD3-stimulated macrophages allows differences to be seen between liposome formulations. Liposomes at 20 μg/ml were incubated with VD3-stimulated macrophages at a final cell density of 1 × 10^6^/ml for 24 h. After incubation with PE-conjugated antibodies, analysis of the co-culture was conducted using flow cytometry to determine the percentage of the cell population to express surface markers and the mean fluorescence intensity (MFI) of the macrophages that had associated with liposomes. Macrophages not exposed to liposomes were used to set the negative and positive discriminator for each surface marker to allow for the effect different liposome formulations had on macrophage surface marker expression to be highlighted. Results shown for n = 4 ± SEM with significant results *P < 0.01, **P < 0.001, ***P < 0.0001 from One-way ANOVA and Tukey’s multiple comparison test.

The results shown in Fig. 5 show the percentage of macrophages positive for the stated surface marker expression upon exposure to different liposome formulations compared to macrophages alone, which has been set as the negative control of ~ 2% positivity. This allows the effect of liposomes on surface marker expression to be highlighted. Results show that the co-stimulatory molecules CD40, CD80 and CD86 are differentially expressed dependent on the liposome formulation the macrophages are exposed to. All three markers, in study 1, were also able to highlight DDA:TDB as the most effective liposome formulation at inducing activation in macrophages in vitro.

Figure 5C shows that all formulations aside from DSPC:TDB and DC-CHOL:TDB induce expression of CD40 above the negative control. DDA:TDB increased expression of CD40 significantly above all other formulations to 50% of macrophages positive. DDA:DSPC:TDB (50%) induced CD40 expression in 20% of macrophages, which is significantly different to the expression seen with DDA:DSPC:TDB and DOTAP:TDB. DDA:TDB also increased CD80 expression the most (Fig. 5a), with 16% of macrophages positive, which was a significant increase from macrophages alone, as well as macrophages exposed to DSPC:TDB and DC-CHOL:TDB. Furthermore CD86 is significantly expressed by DDA:TDB compared to all other liposomes apart from DDA:DSPC:TDB (50%) (Fig. 5b). In all cases DC-CHOL:TDB and DSPC:TDB did not induce expression of co-stimulatory markers above that of macrophages alone.

In this case, expression of MHC II was expected to be increased upon liposome exposure as antigen presentation is required. However the data in Fig. 5 shows no significant difference between MHC II expression with and without exposure to liposomes, ranging from an average of 5% positive cells on macrophages alone to 8% positive cells when exposed to DDA:DSPC:TDB (50%). One reason for this may be that the HLA gene used, HLA-DR, is not always expressed on macrophages, as it has been shown in some cases to only be expressed on THP-1 monocytes and not differentiated THP-1 macrophages^43^. Another reason could be that macrophages are exposed to liposomes that do not contain antigen, the presence of antigen may be required for MHC II transportation to the surface of the macrophage.

Unlike the use of the percentage positive outcome, which shows how many cells within the population were positive for a specific marker, mean fluorescence intensity shows the extent of positivity of the cell fluorescence and therefore indicates the level of expression. Figure 5 shows that the MFI results obtained show a similar trend for each marker as the percentage positive results.

DDA:TDB again produced the highest MFI from CD40 expression of 4900 (Fig. 5g), which was significantly higher than the MFI from the negative control of 1800 and all other liposome formulations. The same was seen with percentage positive results in Fig. 5C, however the number of macrophages positive for CD40 expression when exposed to DDA:DSPC:TDB (50%) was also significantly higher than the control. All liposome formulations failed to induce an MFI from CD80 expression significantly above macrophages alone (Fig. 5e). However DDA:TDB produced the highest MFI result of 336, which was significantly higher than the MFI produced from DSPC:TDB and DC-CHOL:TDB of 161 and 142 respectively.

Unlike the percentage positive results seen with CD86 expression (Fig. 5b), none of the MFI results (Fig. 5f) produced from macrophages exposed to the liposome formulations were significantly different to the control, although the trend was the same with DDA:TDB inducing the highest MFI and DC-CHOL:TDB the lowest. In contrast, MFI from MHC II (Fig. 5h) was only significantly different between DDA:DSPC:TDB (50%), which produced the highest MFI of 452, and DC-CHOL:TDB which produced the lowest MFI of 308, a trend also seen with percentage positive results.

**Figure 6:**
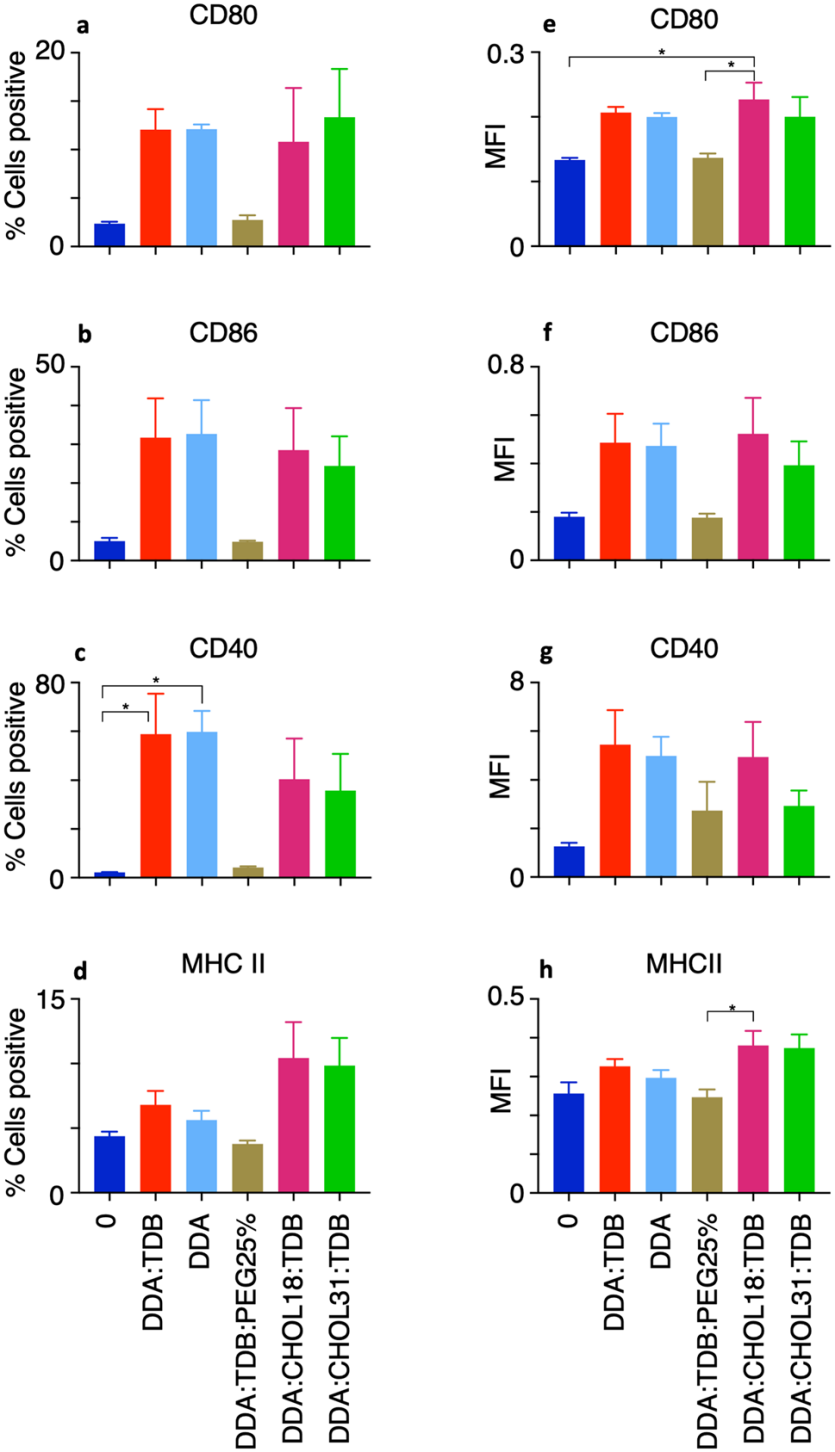
Study 2: Assessing changes in surface marker expression from VD3-stimulated macrophages allows differences to be seen between liposome formulations. Liposomes at 20 μg/ml were incubated with VD3-stimulated macrophages at a final cell density of 1 × 10^6^/ml for 24 h. After incubation with PE-conjugated antibodies, analysis of the co-culture was conducted using flow cytometry to determine the percentage of the cell population to express surface markers. Macrophages not exposed to liposomes were used to set the negative and positive discriminator for each surface marker to allow for the effect different liposome formulations had on macrophage surface marker expression to be highlighted. Results shown for n = 4 ± SEM with significant results *P < 0.01, **P < 0.001, ***P < 0.0001 from One-way ANOVA and Tukey’s multiple comparison test.

When assessing liposome formulations DDA, DDA:TDB:PEG25%, DDA:CHOL18:TDB and DDA:CHOL31:TDB against DDA:TDB in study 2 (Fig. 6e–h), the differentiation between formulations was not as clear as between the formulations in study 1 (Fig. 5). No significant differences were noted for expression of CD86, CD80 and MHC II, however both DDA:TDB and DDA induced CD40 expression significantly above the negative control.

The MFI again gave similar trends to that seen with the percentage positive results, however did highlight further significant differences. DDA:CHOl18:TDB was shown to produce an MFI of ~ 0.2, which was significantly higher than that obtained from the negative control and DDA:TDB:PEG(25%). DDA:CHOL18:TDB also produced a significantly higher MFI for MHC II expression compared to DDA:TDB:PEG(25%), however was not significant to the negative control.

### Macrophages exposed to liposomes release inflammatory cytokines

To establish if further differentiation between the formulations could be highlighted, protein expression of inflammatory cytokines was chosen as another measure to assess activation in macrophages. As, as well as the expression of co-stimulatory markers, which were assessed in Fig. 5 by analysing protein expression on the cell surface, inflammatory cytokines are also produced and released from macrophages upon activation.

Upon activation macrophages are known to release inflammatory and anti-inflammatory cytokines to induce downstream immune responses; cellular infiltration, cell activation and communication, and the anti-inflammatory cytokines are released in order to elicit control over the immune response so chronic inflammation does not occur. It was hypothesised that liposomes that are known to induce an efficient immune response in vivo*,* and also those that induced co-stimulatory marker expression in vitro*,* would be successful at inducing the release of inflammatory cytokines in vitro. Figure 6 assesses cytokine release from macrophages exposed to the 10 different liposome formulation for 24 h.

**Figure 7:**
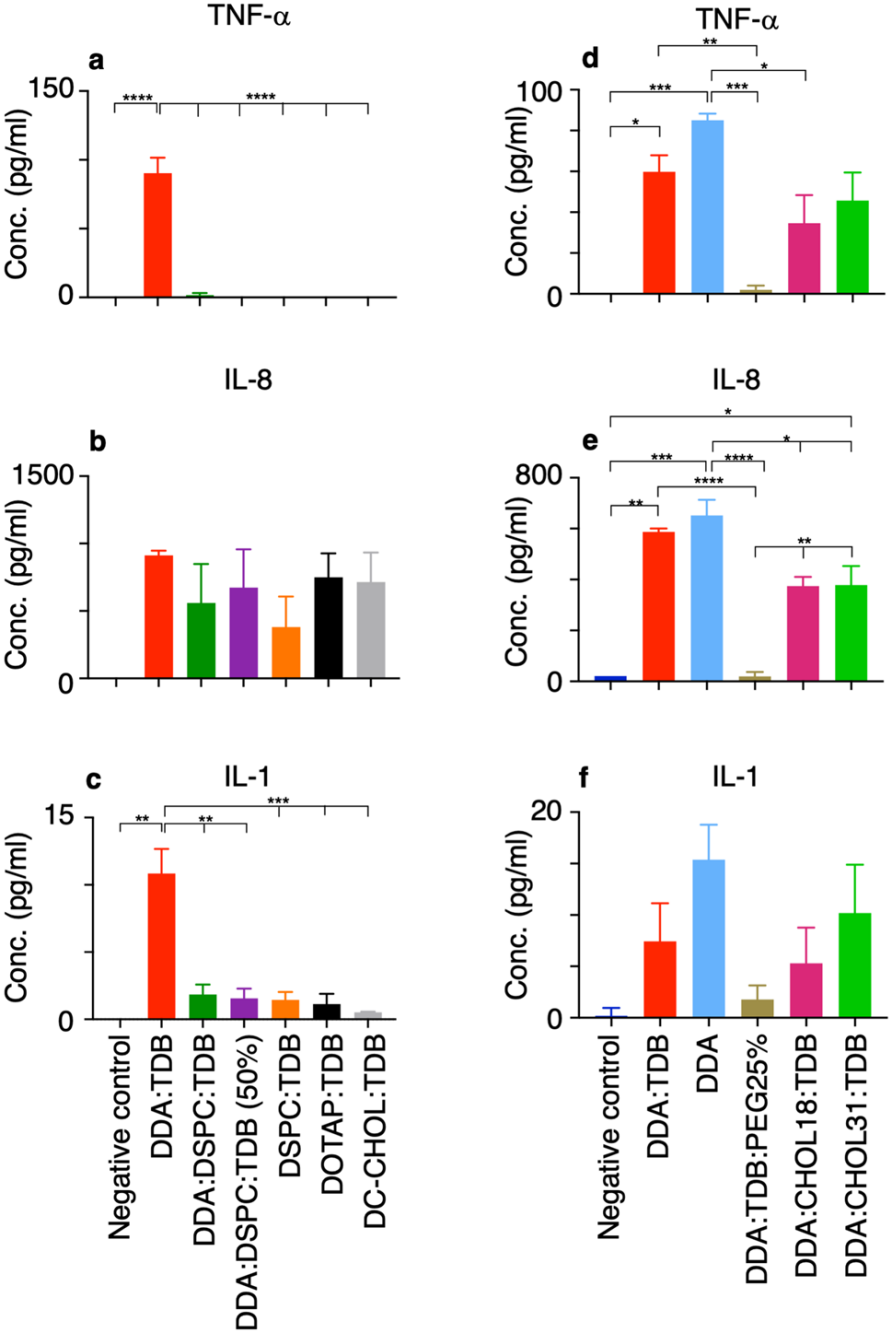
Different liposome formulations vary the level of pro-inflammatory cytokine release from macrophages. THP-1 monocytes were differentiated into macrophages with the addition of 100 nM VD3 and incubated at 37 °C for 48 h. 1 ml of liposomes at 20 μg/ml were added to 1 ml of macrophages at 2 × 10^6^/ml and incubated for a further 24 h. Samples were centrifuged at 300×*g* for 5 min to pellet the cells. The supernatant was then used in ELISA assays to obtain levels of TNF-α (**a,d**), IL-8 (**b,e**), IL-1β (**c,f**). IL-12 and IL-10 results not shown as produced negative concentrations. Negative control = macrophages alone. Results shown for n = 3 (mean ± SEM) with One-Way ANOVA and Tukey’s multiple comparison test, *P ≤ 0.05, **P ≤ 0.005, ***P ≤ 0.0005, ****P ≤ 0.0001.

When comparing DDA:TDB to the other cationic formulations, DOTAP:TDB and DC-CHOl:TDB and the formulations with varying amount of both DDA and DSPC in study 1, TNF-α (Fig. 7a) and IL-1β (Fig. 7c) were the two cytokines that differentiated DDA:TDB as the most effective liposome at inducing cytokine production. DDA:TDB induced the production of 90 pg/ml of TNF-a and 11 pg/ml of IL-1b, whilst all other formulations failed to induce the cytokines to a level above that released from macrophages in a resting state (negative control). IL-8 (Fig. 7b) did not show the same trend, in that all liposome formulations produced a similar amount of IL-8, with no significant difference detected between the IL-8 concentration produced from the negative control and all liposome formulations. However, the negative control produced 0 pg/ml IL-8 and DDA:TDB induced an average production of 912 pg/ml (+ /- 35). The other liposome formulation produced more varied results within a standard error of 200–300 pg/ml.

When investigating the effect of cholesterol and PEG addition into the DDA:TDB formulation and the removal of the immunostimulant TDB, in study 2, the production of TNF-α (Fig. 7d), IL-8 (Fig. 7e), IL-1β (Fig. 7f) all showed a similar trend in how effective each of the liposomes were at inducing cytokine release from macrophages. In all cases, DDA alone and DDA:TDB produced the highest concentration of cytokines, which was unexpected due to the known immunostimulatory activity of TDB. The addition of cholesterol into the DDA:TDB formulation appeared to decrease the efficacy of the liposomes to induce pro-inflammatory cytokine production in macrophages. As well as the addition of PEG to produce DDA:TDB:PEG25%, which failed to induce production of all three inflammatory cytokines significantly above the negative control.

DDA alone and DDA:TDB produced TNF-α concentrations (Fig. 7d) significantly above the negative control. DDA alone produced an average TNF-α concentration of 85 pg/ml (± 3), not significantly different to DDA:TDB that produced an average of 60 pg/ml (± 8). DDA:CHOL18:TDB and DDA:CHOL31:TDB produced an average of 35 pg/ml (± 14) and 46 pg/ml (± 14) respectively, concentrations not significantly different to each other or to the negative control, which could be due to the high variability in results.

For IL-8 production (Fig. 7e) all liposome formulations, except DDA:TDB:PEG25%, produced concentrations significantly above the negative control. DDA alone and DDA:TDB produced the highest concentrations of 652 pg/ml and 587 pg/ml. Again the addition of cholesterol reduced the concentration of IL-8 produced from macrophages to 374 pg/ml when exposed to DDA:CHOL18:TDB and 378 pg/ml (+ /- 75) when exposed to DDA:CHOL31:TDB; concentrations not significantly different from each other but significantly lower than from DDA alone.

As mentioned, IL-1β production (Fig. 7f) followed the same pattern, however due to large error bars the one-way ANOVA did not find any significant differences between the mean concentrations produced from each liposome formulation or the negative control.

### Could cytotoxicity be a cause for macrophage activation?

In vivo, cationic liposomes have shown levels of toxicity at the site of injection, which is proposed as one of the adjuvant mechanisms of CAF01. It was therefore hypothesised that results observed within the in vitro assays could be a result of liposome toxicity to macrophages. With the cell migration assay happening between membranes, with no physical contact between the upper and lower chamber, it could be hypothesised that part of the attraction is due to macrophage apoptosis as they come into contact with liposomes. The macrophages would then produce apoptotic signals that act as chemoattractants for further macrophage migration. Furthermore, the high activation capacity observed with CAF01, could also be due to cellular toxicity, as apoptotic/necrotic signals would activate surrounding cells. To test this hypothesis, liposomes were incubated with macrophages over a 24 h period. Throughout the 24 h duration, samples of the co-culture were taken and stained with AxV and Pi and analysed via flow cytometry for markers of apoptosis and necrosis.

**Figure 8:**
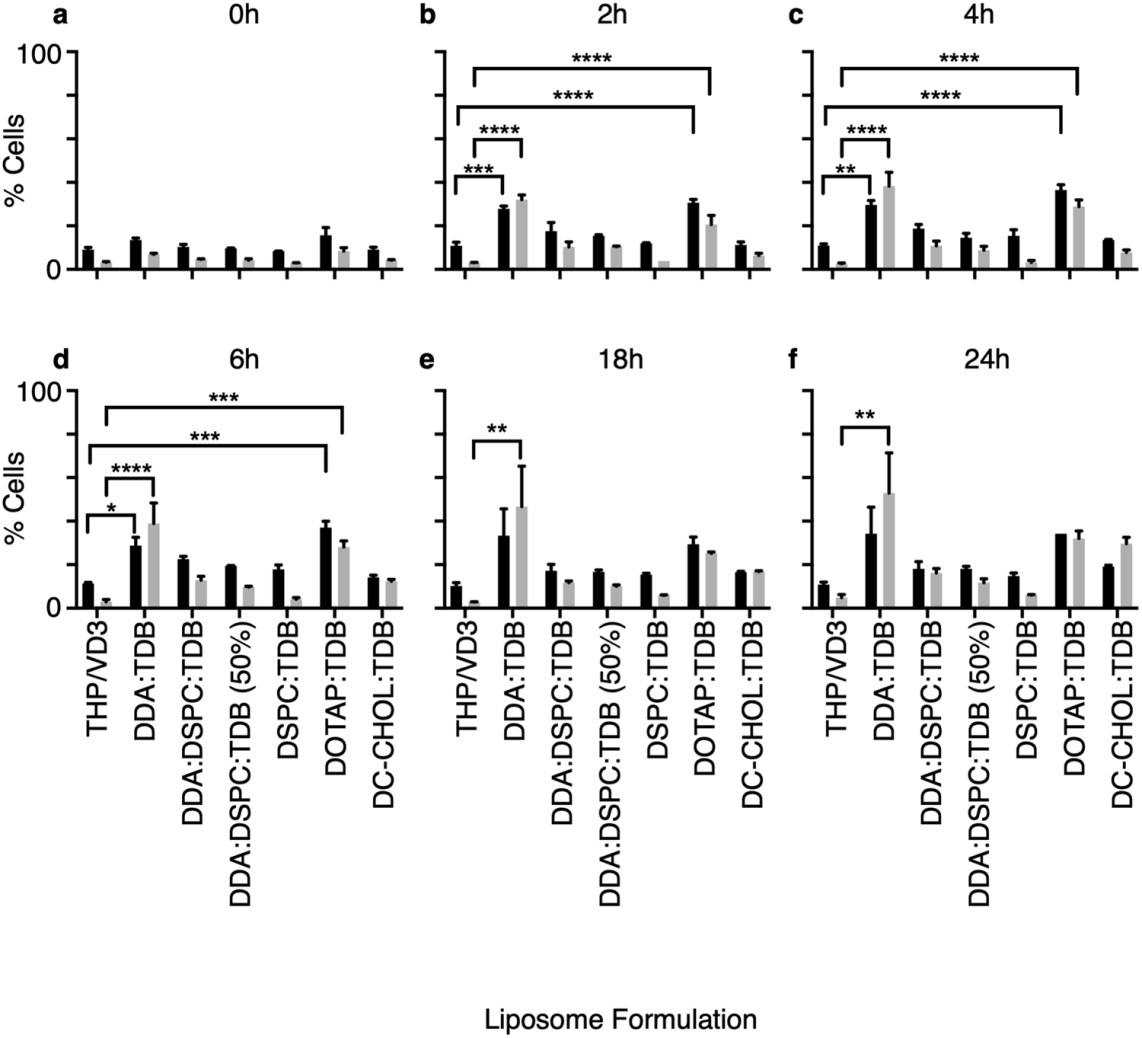
DDA:TDB and DOTAP:TDB significantly hinder macrophage viability within 2 h of exposure. 2 × 10^6^/ml THP-1-derived macrophages were incubated at 37 °C with liposomes at 20 μg/ml, over 24 h. At various intervals, 50 μl of the co-culture was placed in 500 μl AxV binding buffer and stained with 5 μl of both annexin V (AxV-FITC) and propidium iodide (PI-PE). 10,000 events analysed via flow cytometry. Results shown as the percentage of cells positive for AxV-FITC (Black bars) and PI-PE (grey bars) for n = 2 (mean+/- SEM). *P ≤ 0.05, **P ≤ 0.005, ***P ≤ 0.0005 and ****P ≤ 0.0001 with two-way ANOVA with Tukey’s multiple comparison test. Negative control = macrophages alone.

Results show the percentage of cells within the macrophage population that were positive for annexin V and propidium iodide staining, to highlight the possible toxicity of liposomes. Immediately after incubation with liposomes, the percentage of cells positive for AxV and PI were not significantly different to macrophages in the absence of liposomes (Fig. 8a). Within 2 h of incubation 28% of macrophages exposed to DDA:TDB had become positive for AxV, suggesting progression into apoptosis (Fig. 8b). 32% of these cells were positive for PI, which stains cells with a disrupted membrane through necrosis. This was significantly different to macrophages alone, where only 11% of cells were progressing into apoptosis and only 3% were necrotic. The only other liposome to induce cell death significantly above macrophages alone in 2 h, was DOTAP:TDB. DOTAP:TDB, another cationic liposome, caused a 21% increase in macrophages to become positive for AxV and 18% to become positive for PI compared to macrophages alone.

Four hours (Fig. 8c) of exposure to DDA:TDB did not significantly induce more macrophages to progress into apoptosis, as still only 30% of cells were positive for AxV. However, there was a 6% increase in cells that had become positive for PI and progressed into necrosis. At 4 h, 37% of macrophages incubated with DOTAP:TDB were positive for AxV and 29% were now positive for PI. Incubation of macrophages with liposomes for a further 2 h did not change the viable state of the cells (Fig. 8d), it was not until 18 h of incubation where another increase in the percentage of cells progressing into cell death was observed. At 18 h, 34% of macrophages exposed to DDA:TDB were positive for AxV and 47% of cells had progressed to necrosis and were positive for PI. 24 h of exposure to DDA:TDB caused a further increase in cells positive for PI to 53%, however the percentage of cells positive for AxV remained the same. No change in cell viability was seen with DOTAP:TDB for the remaining exposure time.

These results suggest that DDA:TDB and DOTAP:TDB were toxic to macrophages at a concentration of 20 μg/ml, the effects of which happened within 2 h of exposure. The other liposome formulations did not induce cell death in the macrophages significantly above the negative control and were therefore deemed non-toxic.

### In vitro function of liposomes correlates to their in vivo IFN-ү efficacy

The majority of the in vitro functions, independently of each other, highlighted that DDA:TDB was the most efficient liposome formulation at association, migration and activation of macrophages. The in vitro functions also highlighted that a formulation known in vivo to not produce an efficient immune response, like DSPC:TDB, was not efficient at any of the in vitro functions. The other liposome formulations, that were highlighted as ‘++’ intermediate in vivo efficacy in Fig. 1, showed mixed in vitro functions when assessing association, migration and activation independently. In order to see if the in vitro efficacy of liposome formulations could help to predict their in vivo efficacy, it was therefore concluded that a combination of the in vitro functions may be a more accurate prediction, as in vivo, the events of macrophage migration, association and activation all have to happen in order for a successful immune response to be induced against a vaccine.

In order for each in vitro function to be combined to create an overall in vitro strength for each liposome formulation, the in vitro results were taken as a percentage of DDA:TDB, as was conducted when establishing an in vivo strength for each formulation in Fig. 1. By then averaging the percentage of DDA:TDB across all in vitro functions, for each liposome formulation, an in vitro strength of ‘+++’ ≥ 80%, ‘++’ 50–79% and ‘+’ < 50% was established (Table 4).

**Table 4:** An overall in vitro function index differentiates liposome formulations according to their percentage response of DDA:TDB.

Liposome formulations	% of DDA:TDB					
	Migration (16 h)	Association (% positive x MFI) (30 min)	Inflammatory Cytokine Index	Surface Marker Index	Total Average	Overall in vitro strength
DDA:TDB	100	100	100	100	100	+++ > 80%
DDA:DSPC:TDB	61	82	55	34	58	++50–79%
DDA:DSPC:TDB (50%)	101	57	66	38	66	++50–79%
DSPC:TDB	35	0.44	37	31	26	+ < 50%
DOTAP:TDB	63	100	74	50	72	++50–79%
DC-Chol:TDB	13	118	70	19	55	++50–79%
DDA alone	84	91	115	94	94	+++ > 80%
DDA:TDB:PEG25%	4	88	4	4	27	+ < 50%
DDA:CHOL18:TDB	123	86	63	65	86	+++ > 80%
DDA:CHOL31:TDB	97	61	66	36	63	++50–79%

The table shows the percentage response of DDA:TDB for each liposome, for each in vitro function individually and as an overall average. % of DDA:TDB results were calculated for in vitro migration by using the number of macrophages migrated at 16 h, whilst association results at 30 min were used. The inflammatory cytokine index was established by averaging the total of TNF-α, IL-β and Il-8 for each liposome formulation. The surface marker index was produced by calculating the overall expression level for each marker (obtained by multiplying the percentage positive results by the MFI) and then taking an average of CD40, CD80, CD86 and MHC II.

Table 4 highlights the in vitro efficacy of each of the liposome formulations, for all the in vitro functions, both independently and combined. Results presented emphasise the fact that each function gives a slightly different profile of in vitro efficacy for the liposome formulations. Assessing in vitro migration highlighted DDA:TDB, DDA:DSPC:TDB (50%), DDA alone, DDA:CHOL18:TDB and DDA:CHOL31:TDB as liposome formulations with comparable in vitro ability to attract VD3-stimulated macrophages. Whilst it showed DSPC:TDB, DC-CHOL:TDB and DDA:TDB:PEG(25%) as formulations ineffective at inducing macrophage migration. This then contrasted to in vitro association results, where DDA:TDB:PEG(25%) produced a response within 88% of DDA:TDB and categorised as ‘+++’. However the in vitro activation of macrophages by DDA:TDB:PEG(25%) showed the same result as the in vitro migration and again categorised the formulations as ‘+’ in vitro strength. Aside from DSPC:TDB, which showed an in vitro efficacy of ‘+’ for all in vitro functions, the other liposome formulations varied in their in vitro abilities, dependent on the function assessed. This emphasised the requirement to combine the in vitro functions into one in vitro combination index to establish the overall in vitro efficacy of the formulations, as taking the functions individually could bias the results and potentially miss a liposome formulation that would be effective in vivo.

An overall in vitro strength that incorporates all the in vitro functions tested, highlights that DDA:TDB, DDA and DDA:CHOL18:TDB are the most effective liposomes in vitro, all categorised as ‘+++’. It then differentiates DDA:DSPC:TDB, DDA:DPC:TDB (50%), DOTAP:TDB, DC-CHOL:TDB and DDA:CHOL31:TDB from the most effective liposome formulations and categorises these formulations as ‘++’. Furthermore, both DSPC:TDB and DDA:TDB:PEG25% were highlighted as the least effective liposomes in vitro and categorised as ‘+’.

To further emphasise this categorisation of the liposome formulations, principal component analysis was conducted. A biplot of the liposome formulations from both studies, alongside the in vitro functions tested, is shown in Fig. 8. Data for additional surface markers assessed (CD11b, CD16 and CD14) are shown in Supplementary Fig. S5.

**Figure 9:**
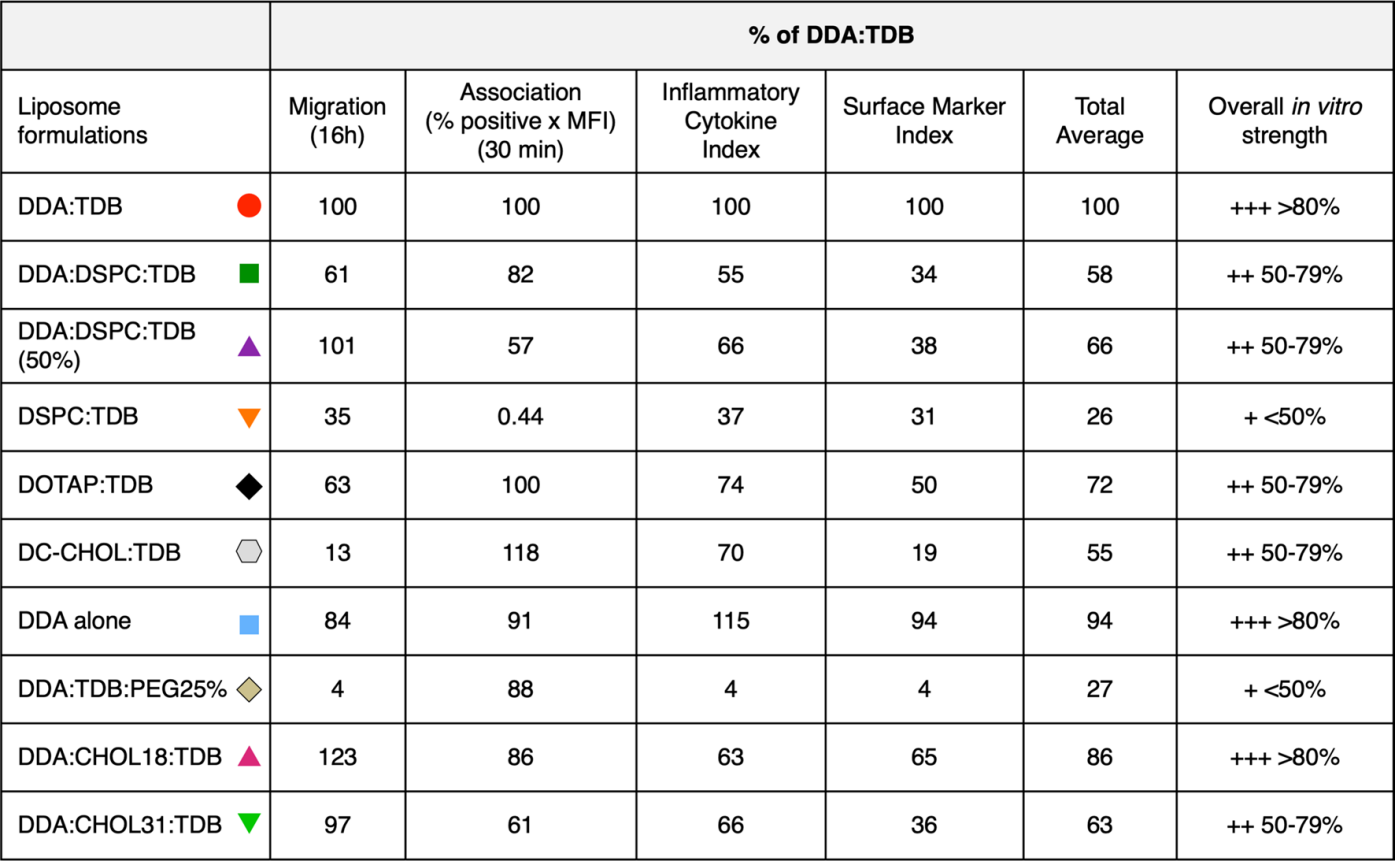
Principal Component Analysis of all 10 liposome formulations and their in vitro functions differentiates DDA:TDB. All 10 liposome formulations and their corresponding in vitro responses were analysed together using principal component analysis and presented on a Biplot. The Biplot shows in vitro functions as loading factors on two principal components (PC1 and PC2) and liposomes are grouped according to functional similarity using PC scores. Ellipses indicate the profile for each liposomes and highlights functional overlap.

Principal Component Analysis (PCA) analysis of all liposome formulations shows DDA:TDB to be differentiated from the other formulations. Results in Fig. 9 appear to be comparable to the in vivo efficacy of the liposome formulations (Fig. 1). DSPC:TDB, DDA:TDB:PEG25%, DC-CHOL:TDB and DOTAP:TDB are all known to be least effective at inducing an in vivo IFN-ү response and are all on the same plane of PC2. Whilst DOTAP:TDB appears to positively correlate CD14 and CD11b, the other three least effective formulations do not correlate with any in vitro function. DDA:DSPC:TDB, DDA:DSPC:TDB (50%), DDA:CHOL18%:TDB and DDA:CHOL31%:TDB show medium effectiveness in vivo and all score around 0 on PC2. Interestingly, both DDA:DSPC:TDB and DDA:DSPC:TDB (50%) are both correlated with CD80, CD16, MHC II, CD14 and CD11b, whilst the formulations containing cholesterol do not appear to correlate positively with any of the in vitro functions. The profile of DDA alone overlaps with DDA:CHOL18:TDB and DDA:CHOL31:TDB and has a PC score of 2. Even though the in vitro assays predicted DDA alone to be as effective as DDA:TDB, the PCA analysis has highlighted in vitro functions that could differentiate the two formulations. DDA alone is most closely correlated with migration, association, IL-1β and TNF- α, whereas DDA:TDB is completely distinguishable from the other formulations and is correlated with CD40, CD86 and IL-8.

This highlights that each of the in vitro assays, both independently and as an in vitro combination index, can differentiate between different liposome formulations. However, it needed to be understood whether this in vitro categorisation of the liposome formulations, correlated to their ability to induce an immune response in vivo. In order to do this, the in vitro strength of the liposome formulations was correlated to the in vivo efficacy established in Fig. 1, from the in vivo production of IFN-ү.

**Figure 10:**
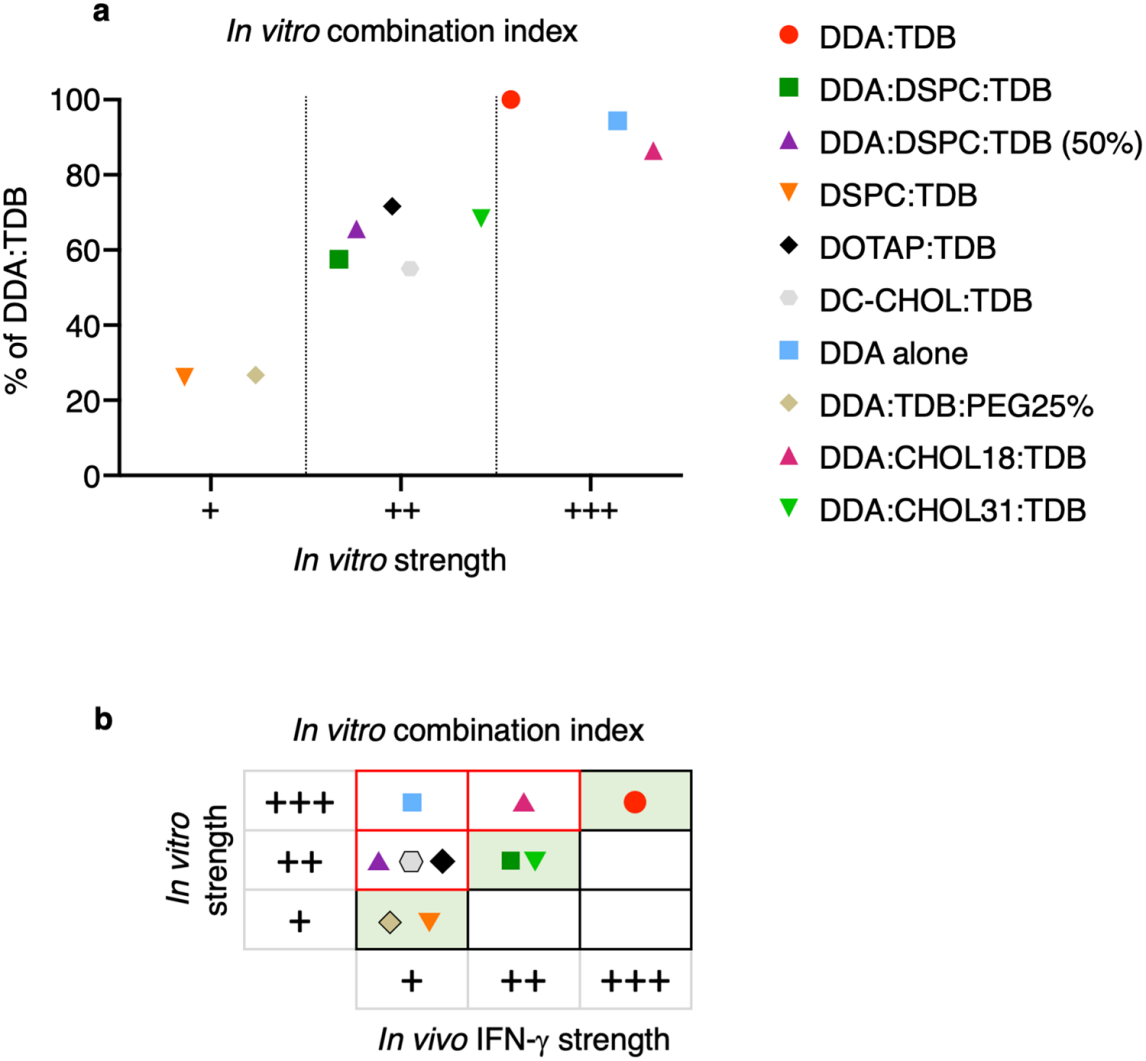
An in vitro combination index consisting of inflammatory cytokines, surface markers, association and migration predicts 5 out of 10 formulations correctly. (**a**) shows the % of DDA:TDB results obtained from the average number of macrophages migrated at 16 h, the in vitro association at 30 min (percentage positive cells X MFI), average co-stimulatory marker expression (in which expression for each individual marker was determined as the product of percentage positive and MFI) and the average cytokine production, categorised as a ‘+++’ ≥ 80%, ‘++’ 50–79%, ‘+’ < 50% in vitro strength. (**b**) Compares the in vitro strength obtained from all in vitro functions (**a**) against the in vivo efficacy of the formulations. Cells shaded in green highlight liposome formulations that were accurately predicted and those with red borders highlight those incorrectly predicted.

Results in Fig. 10a show a graphical representation of the overall in vitro strength of the liposome formulations, highlighting the three distinct categories of ‘+++’, ‘++’ and ‘+’ established from the percentage response of DDA:TDB. The in vivo IFN-ү strength, established in Fig. 1, was then used to allow a comparison to the overall in vitro strength (Fig. 10b). When using all the in vitro functions in combination, 50% of the liposome formulations were accurately predicted for their in vivo efficacy. The in vitro combination index accurately predicted DDA:TDB as an effective liposome formulation in vivo. Furthermore, the in vitro combination index also successfully predicted liposome formulations that are ineffective in vivo at inducing an IFN-y response (DSPC:TDB and DDA:TDB:PEG25%). It also accurately predicted DDA:DSPC:TDB and DDA:CHOl31:TDB as liposome formulations with intermediate efficacy when compared to DDA:TDB. In contrast, DDA and DDA:CHOL18:TDB were over-predicted for their efficacy when using an overall in vitro combination index. If using this in vitro prediction to establish which liposome formulations should progress into further screening in animals, those chosen would be the formulations that show comparable/better results than DDA:TDB, as DDA:TDB is leading formulation within clinical trials. As DDA and DDA:CHOL18:TDB show an in vitro strength of > 80% of DDA:TDB, these two formulations could potentially be chosen to progress into further screening and when using IFN-ү as an in vivo correlate, suggests this would be incorrect.

**Figure 11:**
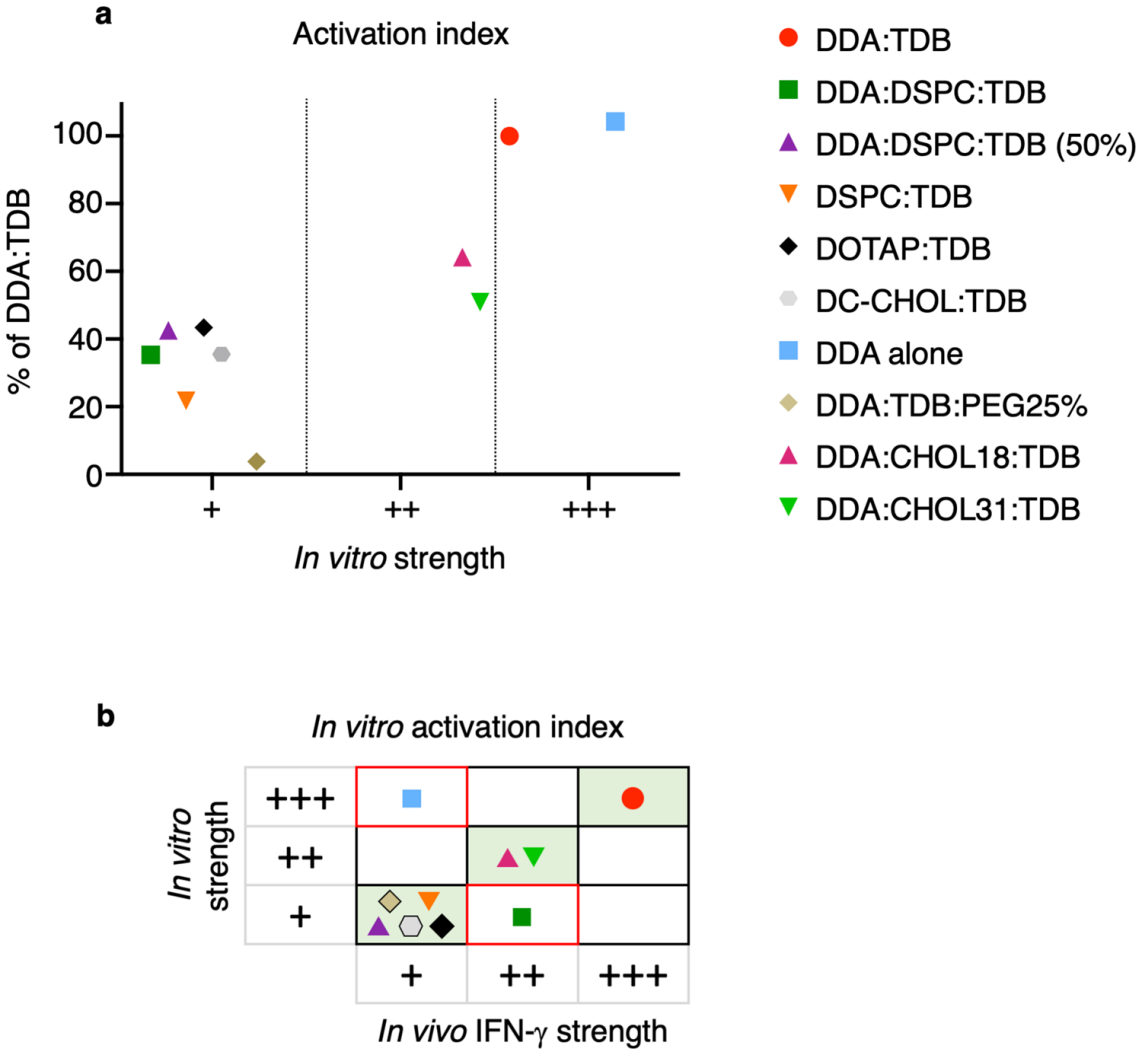
An activation index consisting of inflammatory cytokines and surface markers, CD40, CD80, CD86 and MHC II predicts 8/10 liposome formulations accurately for in vivo efficacy. Figure showing all 10 liposomes and their in vitro strength categorised as a percentage response of DDA:TDB, where ‘+++’ ≥ 80%, ‘++’ 50–79%, ‘+’ < 50%. (**a**) shows liposomes categorised using the in vitro activation index consisting of an average of CD40, CD80, CD86 and MHC II (in which expression for each individual marker was determined as the product of percentage positive and MFI) and TNF-α, IL-8 and IL-1β. The table in (**b**) shows the liposomes’ in vitro strength obtained from the activation index in (**a**) against the in vivo IFN-ү strength.

It was then hypothesised that because IFN-ү production would require the activation of APCs, that an in vitro activation index may be a more accurate predictor of in vivo IFN-y production. Therefore the Fig. 11 shows the in vitro strength results of the liposome formulations, when only using the in vitro ability to induce co-stimulatory marker expression and cytokine production.

As hypothesised, obtaining an in vitro strength using only the expression of co-stimulatory markers and cytokine production accurately predicted more of the liposome formulations for their known in vivo efficacy. The only 2 formulations that were incorrectly predicted, where DDA:DSPC:TDB and DDA alone. DDA:DSPC:TDB now gave an in vitro strength response of ‘+’ instead of ‘++’ and DDA alone still gave a response of ‘+++’. When using the cut off of 80%, for those formulations that would be progressed into further screening, using the activation index means only 1 formulation (DDA alone) would progress further incorrectly, instead of the 2 that would have been predicted when also using in vitro migration and association.

## Discussion

Here we have revealed a suite of in vitro assays and their comparison to known in vivo responses, creating the opportunity for developing a rapid screening tool for development of novel liposome formulations and potentially other vaccine adjuvant candidates, such as lipid nanoparticles. In these assays we reveal that a suite of two tests that assess in vitro activation of macrophages, through co-stimulatory marker expression and inflammatory cytokine release, could be used to predict correctly up to 80% of in vivo responses (when measuring IFN-ү as a correlate of immunity). More importantly the assays were able to accurately distinguish DDA:TDB as the most effective liposome formulation and highlighted the ability to predict those liposomes that would be ineffective in vivo.

Given the PCA analyses, it is reasonable that these assays could be refined even further, if the aim is to find liposome formulations at least as effective as DDA:TDB, then markers such as CD40, CD86 and IL-8 would be the most important to use. However, the PCA analysis also highlights the importance of in vitro association, migration, TNF-α and IL-1β in differentiating between effective liposomes that may have different in vivo functions (i.e. DDA:TDB and DDA alone). Therefore when taking the model forward to other novel existing in vivo data sets for further testing, it may be important to include the whole suite of in vitro assays, considering the different outputs obtained from each function individually. The output may be a streamlined suite that may be used not only in novel liposome formulation development, but could also be used in the wider field of vaccine development. By using a human monocytic cell line we have been able to create a suite of in vitro assays with the target cells of human vaccination in mind, future work could pursue a wider vaccination field by changing the target cell used within the assays. Although dendritic cells are known to be the most important antigen presenting cell in communicating with the adaptive immune response, THP-1 macrophages are easier and quicker to differentiate in culture and have proved their effectiveness within the in vitro predictive model. Furthermore, the assays developed here use a Th1 correlate of immunity, future work could seek to repeat this system looking at other correlates of immunity, e.g. Th2 correlates of immunity and also investigate the addition of T cell models to potentially increase the predictive power of an adaptive immune response.

Efficacy testing of liposome formulations for use as adjuvanted-vaccine delivery systems is mostly conducted in animals, alongside the antigen the vaccine is aimed at. To obtain a set of in vivo data from mice for a single liposome formulation, can take at least 3 weeks, dependent on the immunisation protocol used, and leads to the culling of the animal. The still current prevalence of infectious diseases that do not have a successful vaccine against them and also the emergence of new pathogens, makes it critical to develop ways to speed up the vaccine development process. The in vitro pre-screen allows refined targeting of potentially effective liposome formulations that can then go on into further screening to include the target antigen. To emphasise the power of the in vitro predictive model, a number of the in vitro assays have been used by a research laboratory at the University of Strathclyde during screening of novel liposome formulations. This collaboration has led to a reduction in the use of 200 mice and hopefully continued collaboration with this research laboratory sees the in vitro predictive model fully incorporated into their liposome development and screening process.

## Conclusion

Overall, 80% of liposomes were predicted accurately for their in vivo efficacy when comparing to the in vivo induction of IFN-γ, therefore demonstrating the potential use of an in vitro pre-screen in the development of novel liposome formulations. An in vitro pre-screen could reduce the number of mice used within pre-clinical studies and speed up the development and screening of novel liposome formulations. In conclusion, the data presented demonstrates the use of a set of in vitro assays, which use THP-1-derived macrophages as the model antigen presenting cell, to assess the ability of novel liposome adjuvants to attract, associate and activate macrophages. The combination of the ability of liposomes to induce CD80, CD86, CD40, MHC II expression and release of TNF-α, IL-8 and IL-1β predicted the most liposome formulations correctly, however PCA analysis indicated the potential refinement of the suite of in vitro assays and also the power of including all in vitro functions to differentiate between the efficacy of liposome formulation effectively.

## Supplementary Information

Supplementary Information.
