# Supplementary material for: Development of a rapid in vitro pre-screen for distinguishing effective liposome-adjuvant delivery systems
Source: Sci Rep. 2022 Jul 20;12:12448. doi: 10.1038/s41598-022-14449-7 (PMC9299755; doi:10.1038/s41598-022-14449-7)
Supplement: Supplementary file 1 — Supplementary Information. [file 41598_2022_14449_MOESM1_ESM.pdf]

Supplementary Information:

Development of a rapid *in vitro* pre-screen for distinguishing effective liposome-adjuvant delivery systems

Laura A. J. Feather, Vinod Nadella, Elisabeth Kastner, Yvonne Perrie, Anthony Hilton and Andrew Devitt

Supplementary Data

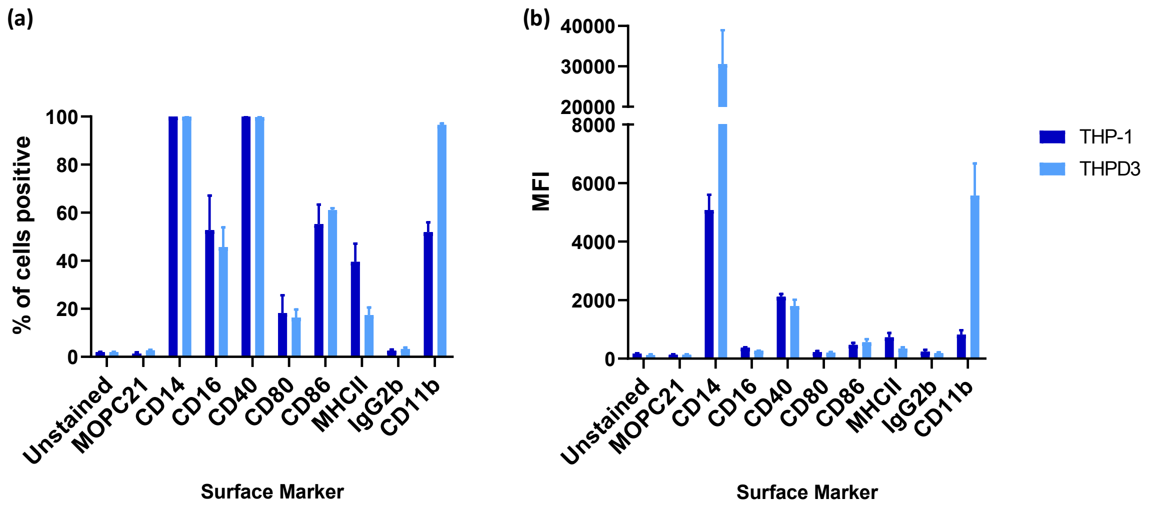

Supplementary Figure 1: Evidence of macrophage maturation from Vitamin D3 stimulation of THP-1 monocytes. THP-1 monocytes and VD3-differentiated macrophages (THP/D3) at a final cell density of $1x10^6$/ml were stained for surface expression of CD14, CD16, CD40, CD80, CD86, MHC II and CD11b with PE-conjugated antibodies. Analysis was conducted using flow cytometry to determine surface marker expression via percentage of cells positive for marker expression **(a)** and MFI **(b)**. Results shown for n=3 (mean +/- SEM).

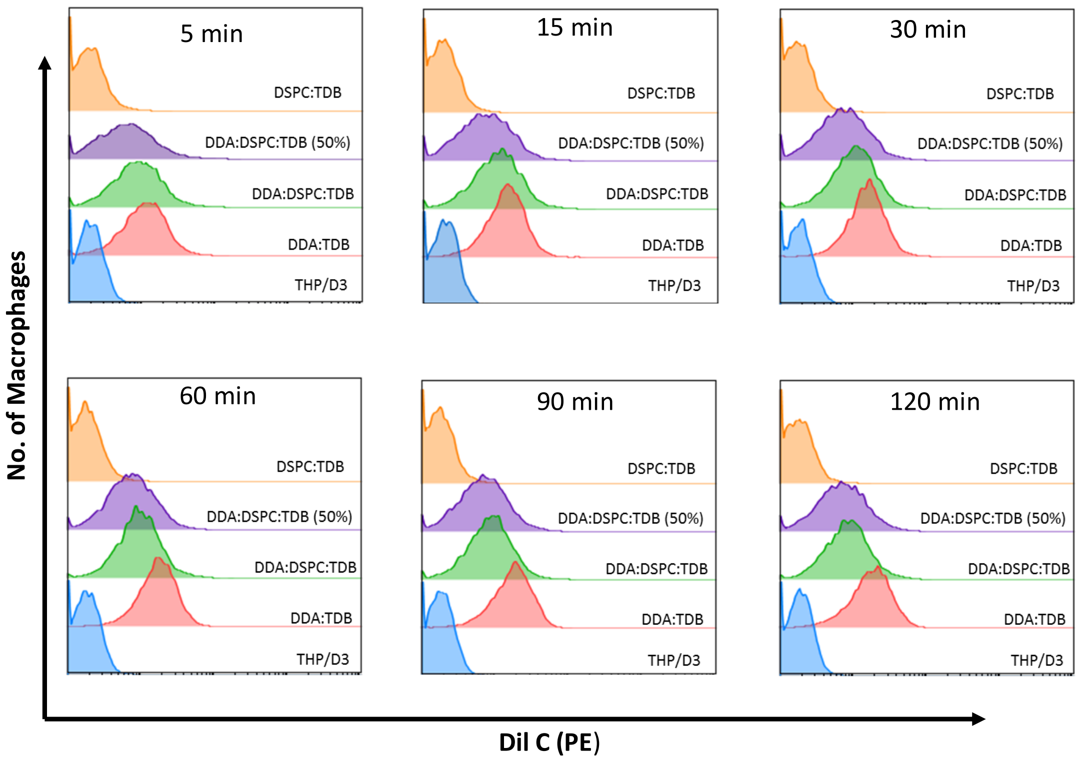

Supplementary figure 2: Flow histograms illustrate the association of fluorescently-labelled liposomes by THP-1 derived macrophages over 2 hours. Flow histograms are shown for macrophages that have associated with each of the liposome formulations (study 1) at 5, 15, 30, 60, 90 and 120 minutes. The peaks in blue represent macrophages in the absence of liposomes (negative control) and the coloured peaks represent macrophages incubated with each liposome formulation. Any shift to the right of the negative control indicates liposome positive cells.

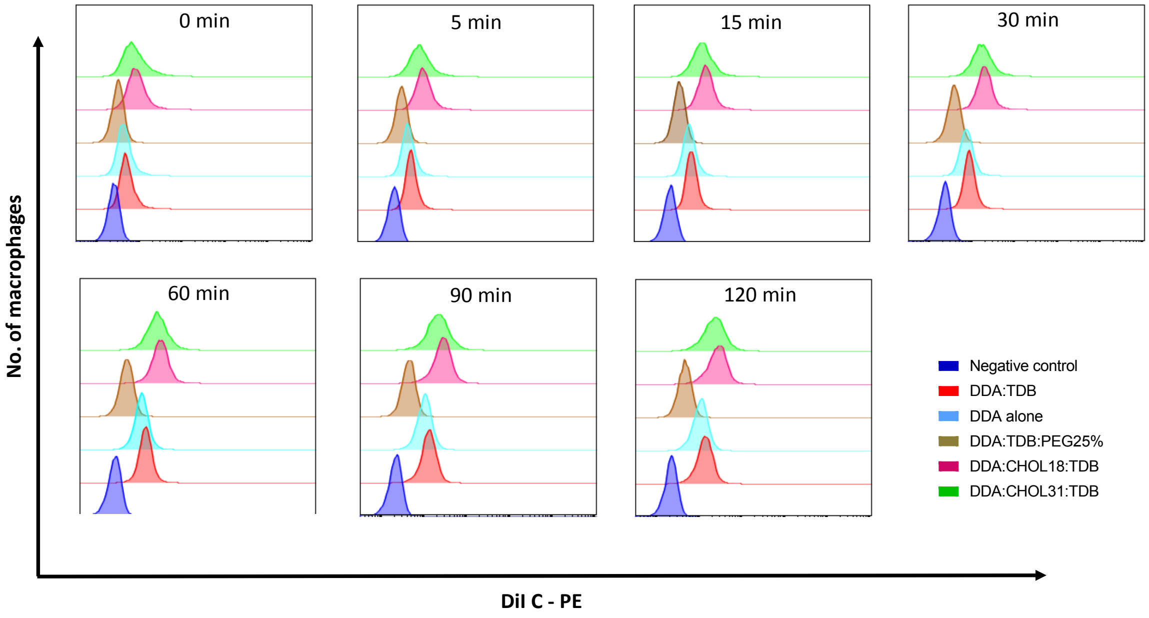

Supplementary figure 3: Flow histograms illustrate the association of fluorescently-labelled liposomes by THP-1 derived macrophages over 2 hours. Flow histograms are shown for macrophages that have associated with each of the liposome formulations (study 2) at 5, 15, 30, 60, 90 and 120 minutes. The peaks in blue represent macrophages in the absence of liposomes (negative control) and the coloured peaks represent macrophages incubated with each liposome formulation. Any shift to the right of the negative control indicates liposome positive cells.

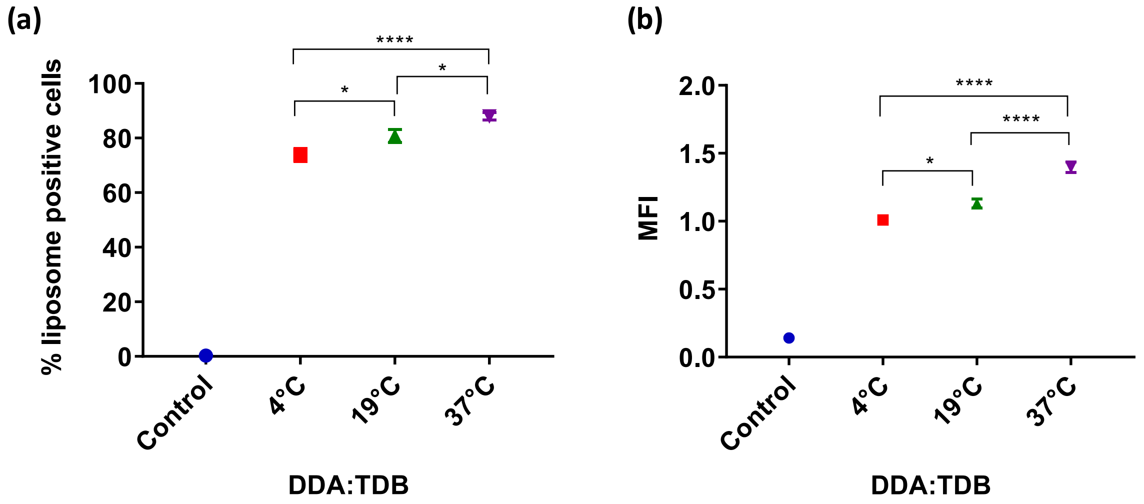

Supplementary figure 4: Association studies at 37°C, 19°C and 4°C suggest influence of electrostatic interactions in liposome-macrophage association. Fluorescently-labelled DDA:TDB liposomes were incubated with THP-1 derived macrophages at a lipid concentration of 20 µg/ml and a cell concentration of $2x10^6$/ml for 30 min, at either 4°C, 19°C and 37°C. 200 µl of co-culture was placed in 200 µl ice cold serum-free RPMI before flow cytometric analysis of 10000 events. **(a)** Shows the percentage of macrophages to have associated with DDA:TDB and **(b)** shows mean fluorescence intensity of cells positive for liposome interaction. Control = THPD3 alone. Data shown for n=3 (mean +/-SEM) with one way ANOVA and Tukey's multiple comparison test, *P≤0.05, ****P≤0.0001.

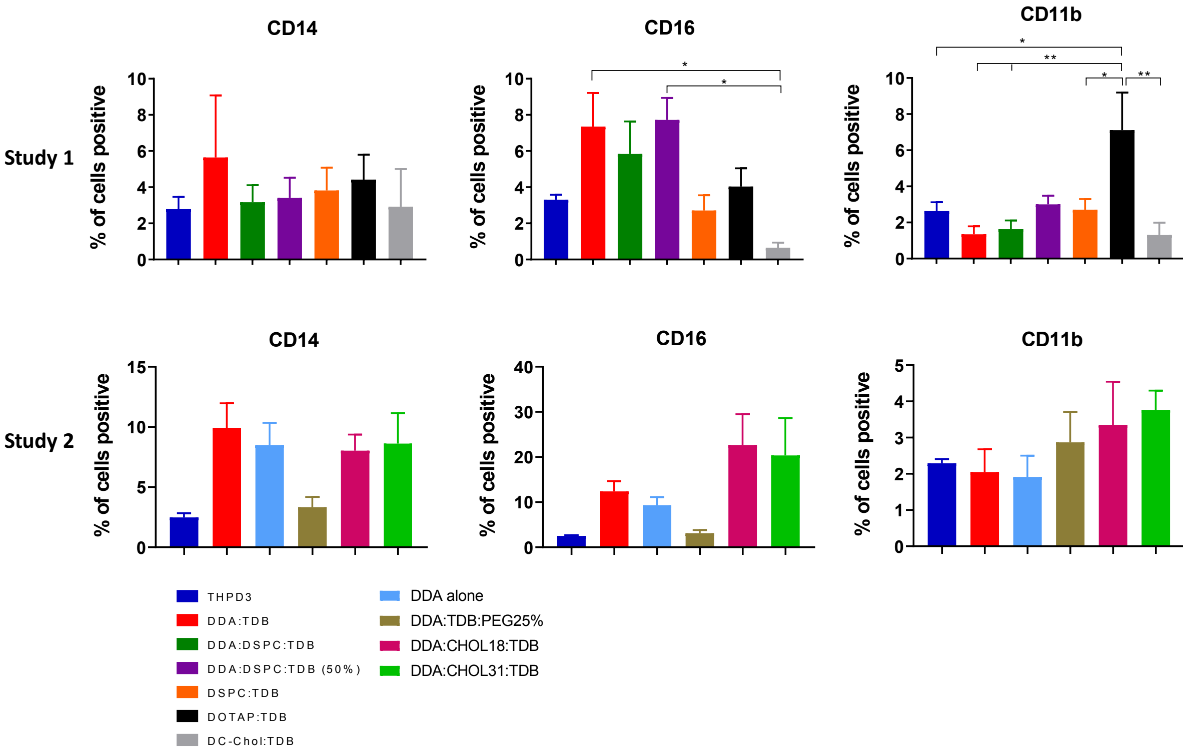

Supplementary figure 5: Induction of surface markers CD14, CD16 and CD11b from macrophages exposed to liposome formulations. Liposomes at 20 µg/ml were incubated with VD3-stimulated macrophages at a final cell density of 1x10^6/ml for 24 h. After incubation with PE-conjugated antibodies, analysis of the co-culture was conducted using flow cytometry to determine the percentage of the cell population to express surface markers. Macrophages not exposed to liposomes (THPD3) were used to set the negative and positive discriminator for each surface marker to allow for the effect different liposome formulations had on macrophage surface marker expression to be highlighted. Results shown for n=4 +/- SEM with significant results *P<0.01, ** P <0.001, *** P <0.0001 from One-way ANOVA and Tukey's multiple comparison test.
